# The rise of Indian summer monsoon precipitation extremes and its correlation with long-term changes of climate and anthropogenic factors

**DOI:** 10.1038/s41598-022-16240-0

**Published:** 2022-07-14

**Authors:** Renaud Falga, Chien Wang

**Affiliations:** Laboratoire d’Aérologie, University of Toulouse III – Paul Sabatier, Toulouse, France

**Keywords:** Environmental sciences, Climate sciences, Atmospheric science, Climate change

## Abstract

The trends of extreme precipitation events during the Indian summer monsoon measured by two different indicators have been analyzed for the period of 1901–2020, covering the entire India in 9 regions segregated by a clustering analysis based on rainfall characteristics using the Indian Meteorological Department high-resolution gridded data. In seven regions with sufficiently high confidence in the precipitation data, 12 out of the 14 calculated trends are found to be statistically significantly increasing. The important climatological parameters correlated to such increasing trends have also been identified by performing for the first time a multivariate analysis using a nonlinear machine learning regression with 17 input variables. It is found that man-made long-term shifting of land-use and land-cover patterns, and most significantly the urbanization, play a crucial role in the prediction of the long-term trends of extreme precipitation events, particularly of the intensity of extremes. While in certain regions, thermodynamical, circulation, and convective instability parameters are also found to be key predicting factors, mostly of the frequency of the precipitation extremes. The findings of these correlations to the monsoonal precipitation extremes provides a foundation for further causal relation analyses using advanced models.

## Introduction

The amount and distribution of precipitation during the Indian summer monsoon (ISM) have a substantial impact on the region’s agricultural systems and thus the livelihood of more than a billion people^1^. These climatological parameters have high interannual and interdecadal variabilities^2,3^ and a part of these could be explained by natural climate variability. Nevertheless, there is a high probability that man-made global or regional climate changes could have also affected these quantities with an extent yet to be examined^4–8^. While the overall ISM rainfall is believed to have decreased during the twentieth century^9^ then reversed since the turn of this century^10^, it has been indicated that the extreme precipitation events might have been rising in some parts of India^11,12^, with hypothesized causes ranging from urbanization^13–16^, increase in dew point temperature^17,18^, to climate variability^19,20^. These hypotheses, as indicated in a recent review by Singh et al. (2019)^21^, were largely proposed based on comparing the trends of extremes with that of a single isolated explanatory variable and thus tended to disagree with each other. Understanding the variation alongside the causes of these extreme events is essential not only for predicting future climate change, but also for making effective mitigation strategies. Here, by applying advanced data science methods in analysing more than a century long surface rain gauge data as well as best available data for other meteorological and climatological variables, or land-use and land-cover (LULC) changes, it has been demonstrated that extreme precipitation events have been increasing in most regions of India, and that such an increase appears to be closely correlated with the long-term changes of certain climatological factors caused by anthropogenic forcing.

## Results and discussion

### Definition of the climatologically homogeneous study regions using a hierarchical clustering method

**Figure 1:**
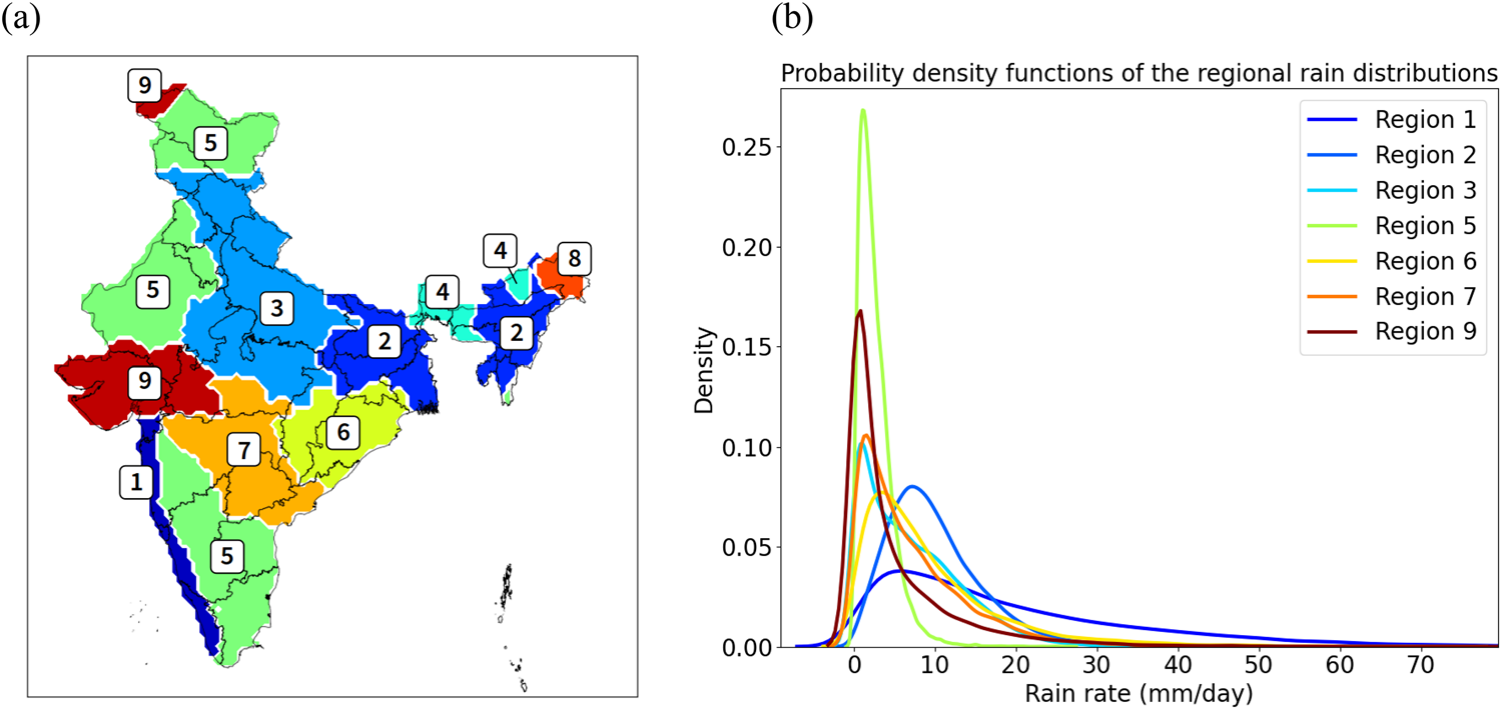
Region clustering. (**a**) The nine analysis regions identified by applying Ward’s minimum variance clustering method to the daily rainfall data in the period of 1901–2020 (every monsoon day during this period has been considered for the clustering), and (**b**) the resulting regional rain distributions in the seven study regions.

Precipitation extremes are low probability events of relatively small spatial scale occurring unevenly across India^22,23^. For this reason, a trend analysis should be ideally performed to an optimal number of regions covering the entire India, each with similar climatic rainfall characteristics to derive suitable regional thresholds for extremes and thus consistent trends, as well as to identify potentially unique driving factors. Following this principle, the trends of two threshold-based rainfall extreme indicators, i.e., the frequency and the intensity (see Methods), have been derived using the Indian Meteorological Department (IMD) high-resolution rainfall gridded dataset, arguably the best available dataset for the purpose. To define relevant study regions, we applied Ward’s minimum variance clustering method to the daily rainfall dataset and found it to be optimal to segregate India into nine different climatologically homogeneous regions (Fig. 1, also “Methods” section). In addition to ensure the suitability of applying the regional thresholds to define the extreme events, this segregation also allows our analysis to better reflect the heterogeneous nature of extreme events across India, as we perform a multivariate regression analysis within each region. The rainfall distributions of these nine regions (Fig. 1b) proves that their climatic conditions are indeed relatively distinct. Note that how to perform spatial averaging to define extremes still lacks a widely accepted solution. Among the previous works, some had chosen to focus on a single region, often being central India^11,12,24^, while others studied the whole India by separating the country into four to six arbitrarily defined regions^20,25^. Differing from these approaches, here we use a statistical method, i.e., cluster analysis, to quantitatively segregate the whole India into different regions, each containing clear internal similarity while displaying substantial difference with others in terms of precipitation characteristics. Understandably, our approach in segregation leads to a higher number of regions compared to what was previously done (e.g., nine regions versus five regions in general). As shown in the following discussion, derived trends of extreme events in our study are similar to those in some previous analyses^25^ over certain regions that largely overlap with those in the latter works. Nevertheless, due to the fact that several areas with unique precipitation characters were aggregated into the same region in the latter works, the derived trends of regional extreme events could thus entitle to some issues. In contrast, due to the quantitative segregation method used in our study, our trend analysis allows us to assess the precipitation characteristics in certain previously unstudied, yet climatologically interesting regions, such as our coastal region 1, a critical zone of monsoon onset rain belt.

In two out of the nine above-defined regions (regions 4 and 8), the confidence in the precipitation data was not sufficiently high to perform the trend analysis, owing to the number of included recording stations in the IMD dataset being either too low or too fluctuating during the considered period. It has been suggested that the varying numbers of stations included in the IMD dataset from year to year could cause certain issues in deriving long-term rainfall trends^10,26^. Indeed, there was a sharp increase in the number of included stations for regions 4 and 8 during the 1970’s, potentially affecting our effort of defining extreme events there (Supplementary Material, Fig. S1).

However, in the seven remaining regions, the number of recording stations, although not exactly constant throughout the century, has not experienced a sharp increase, and this provides us with more confidence when performing the trend analysis using the data of these regions. Furthermore, to ensure the validity of our results, we have performed an additional regional trend analysis only on the grid cells of the seven remaining regions where the number of recording stations is stable throughout the century (Supplementary Material, Fig. S2). The trends derived in the additional analysis are very similar to those derived using all the grid cells in the regions, suggesting that the numerous increasing trends calculated are not due to some statistical artifact induced by an increasing number of recording stations. As a result, our analyses are focused on these seven remaining regions.

### Trend analysis results

The frequency of extreme events has been calculated by considering the daily gridded rainfall dataset. After defining the regional threshold as the 99th percentile of the local monsoon rainfall distribution, the number of grid cells where the daily rainfall exceeded this threshold has been counted for all the monsoon seasons during 1901–2020. The average intensity of such events has also been calculated.

**Figure 2:**
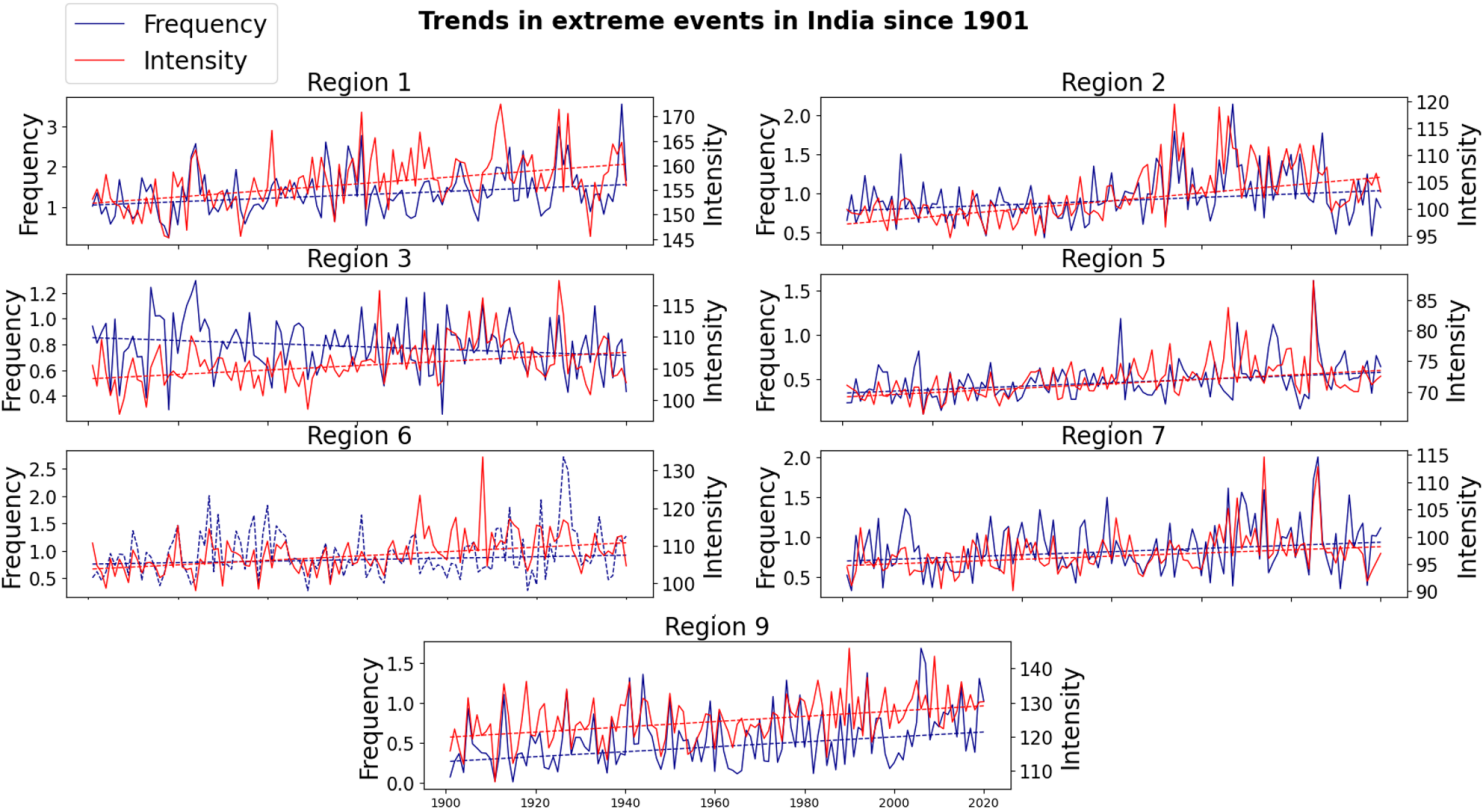
Trends of extreme events. Trends in frequency (blue) and intensity (red) in each region. The frequency of extremes is given in number of extreme events per monsoon season per grid cell, while the intensity is given in mm/day. The time series plotted in dashed line (frequency in region 6) correspond to a statistically insignificant trend (found by performing Mann–Kendall test at the 95% confidence level).

Increasing trends in the intensity of extreme precipitation events have been found in all the seven regions by using Mann–Kendall trend test, for analysis performed on a 5% statistical significance level. Using Sen’s slope estimator, the rising percentages ranging from + 4% in region 7 to + 10% in region 2 have been identified (Fig. 2).

For the frequency of extreme precipitation events, we have identified increasing trends in regions 1, 2, 5, 7 and 9, or the entire west and southwest plus a part of northeast portion of India. Since the number of annual extreme events in a region is dependent on the size of the region, to have comparable results across all regions, we divided the regional number of extremes by the number of grid cells for each region. The rising percentages in frequency in the increasing regions range from + 33% in region 7 to + 134% in region 9. The regions that have witnessed the higher increase are all located in western India, with regions 1, 5 and 9 respectively showing a + 48%, + 68% and + 134% increase in the frequency of extreme events. Apart from the increasing trends in above regions, we have also found a statistically significant decreasing trend in region 3 (north-central India), and a statistically insignificant increasing trend in region 6 in the east coast.

To summarize, among the total 14 trends derived here using two different indicators in seven regions, we have identified 12 statistically significant (95% confidence level) increasing trends and one significant decreasing trend. The remaining trend (frequency in region 6) showed increasing signs but did not pass the Mann–Kendall trend test. These results indicates that the extreme precipitation events during the ISM seasons have increased evidently across majority of India since 1901.

### Breaking point in the middle of the twentieth century

In most derived rising trends particularly of the intensity of extremes, there is a sharper increase in the second part of the twentieth century compared to the first part. Certain previous studies also found a similar difference between the pre-1950 and post-1950 trends and attributed it to urbanization^16^. Indeed, India experienced an intense urbanization between the 1950s and 1980s. The other LULCs such as agricultural lands (particularly the croplands), grasslands and forests were also modified substantially during this period^27^. Such an anthropogenic factor is believed to have an important impact on climate and rainfall^28^, implying that the LULC changes could potentially amplify the rising trends of precipitation extremes in the second part of the twentieth century. To verify this difference in the trends, we have applied a running slope difference (RSD) t-test, a statistical method designed to detect the trend turning in a time series^29^. We find that among the 12 statistically significant increasing trends, 10 of them display a statistically significant breaking point between 1940 and 1970, suggesting that the anthropogenic forcing resulted from man-made LULC changes might have had impact on the trends. The breaking points in the time series are even more sharp when looking at the trends in intensity.

### Random forest multivariate regression

Using an ensemble multi-variate and nonlinear machine learning technique, i.e., random forest^30^, the potential predictors behind the above-discussed evolution of ISM precipitation extremes since 1901 have been further analyzed with a synergy of best available data of LULC changes and certain climate variabilities. Firstly, 120 year-long multivariate input data have been used to fit a random forest regression model against observations of extreme event indicators. Furthermore, using the successfully trained random forest regression model and a feature importance functionality, we were able to determine the most responsible variables, or effective predicting features, to the rise of extreme events. A major purpose of this practice is to identify the correlations between long-term (century) evolutions of certain climatological (rather than episodic) factors or features and the observed climate trends of the ISM precipitation extremes, thus providing leads for additional attribution analyses should additional data were made available and most importantly, for using advanced models to further examine the causal relations between certain effective predicting features and the observed extreme event trends.

Note that random forest is a nonlinear regression algorithm, meaning that unlike classical linear regression, it can find complex nonlinear correlations between the input features and the output result. Hence, it is better suited for complex systems like the climate system with different feedbacks. For example, an input parameter such as sea surface temperature might have had an important positive influence on the extreme events for a certain period of time, while at some other period, another parameter (e.g., the circulation) could become more influential. Random forest regression model can capture this type of behavior, and its feature importance tool also gives us a good insight on the variables that had the most impact on the trends throughout the whole time period.

The feature importance has been calculated using the conditional permutation method in order to determine the contribution percentages of each given feature in predicting the accurate values of the different extreme events indicators (Methods). Traditionally, the feature importance is calculated using either the Gini impurity decrease or the classical permutation importance. However, the presence of correlated input features, which is often the case with meteorological data, has been shown to possibly impact the ability of both methods to identify strong predictors^31^. These classical feature importance methods can still correctly rank the driving features of the trends of extreme events^32^ (ordinarily called true predictors), but they might likely lead to overestimating the importance of some alternative features that are correlated to the true predictors^33^. Therefore, we chose to calculate the conditional feature importance (Strobl et al., 2008)^33^, which can better reflect the true impact of each predictor variable on the different trends, even in the presence of highly correlated features. This allows us to have a high confidence in the derived variables’ contribution through the feature importance. In addition, we also performed feature selection (Methods) in order to keep only the features showing high contributions to the prediction using random forest model.

Note that this feature importance only reflects the contribution of each feature to the prediction of the testing dataset (Methods). For example, a conditional permutation importance of 50% for the urban fraction does not necessarily mean that 50% of the increase in extreme events was actually due to the increase in urban fraction, but rather represents the importance of the urban fraction in the random forest model prediction of extremes. Nevertheless, by including a considerable number of features arguably for the first time, our analysis still represents a step forward from previous works using only single or a few factors. In fact, the regression scores of 13 out of 14 regressions are very good, with accuracy ranging from 0.68 to 0.96 (Fig. S3), suggesting that our model succeeds in predicting the extreme events using our input data with a good confidence. Therefore, a feature showing a high contribution to the regression model is likely to be an effective cause for the observed trends. The only regression showing a low score is the frequency trend in region 3 (R^2^ = 0.39), meaning that we did not manage to find accurate predictors for this particular trend. Consequently, we do not show the feature importance results for this trend.

### Choice of the input features

**Figure 3:**
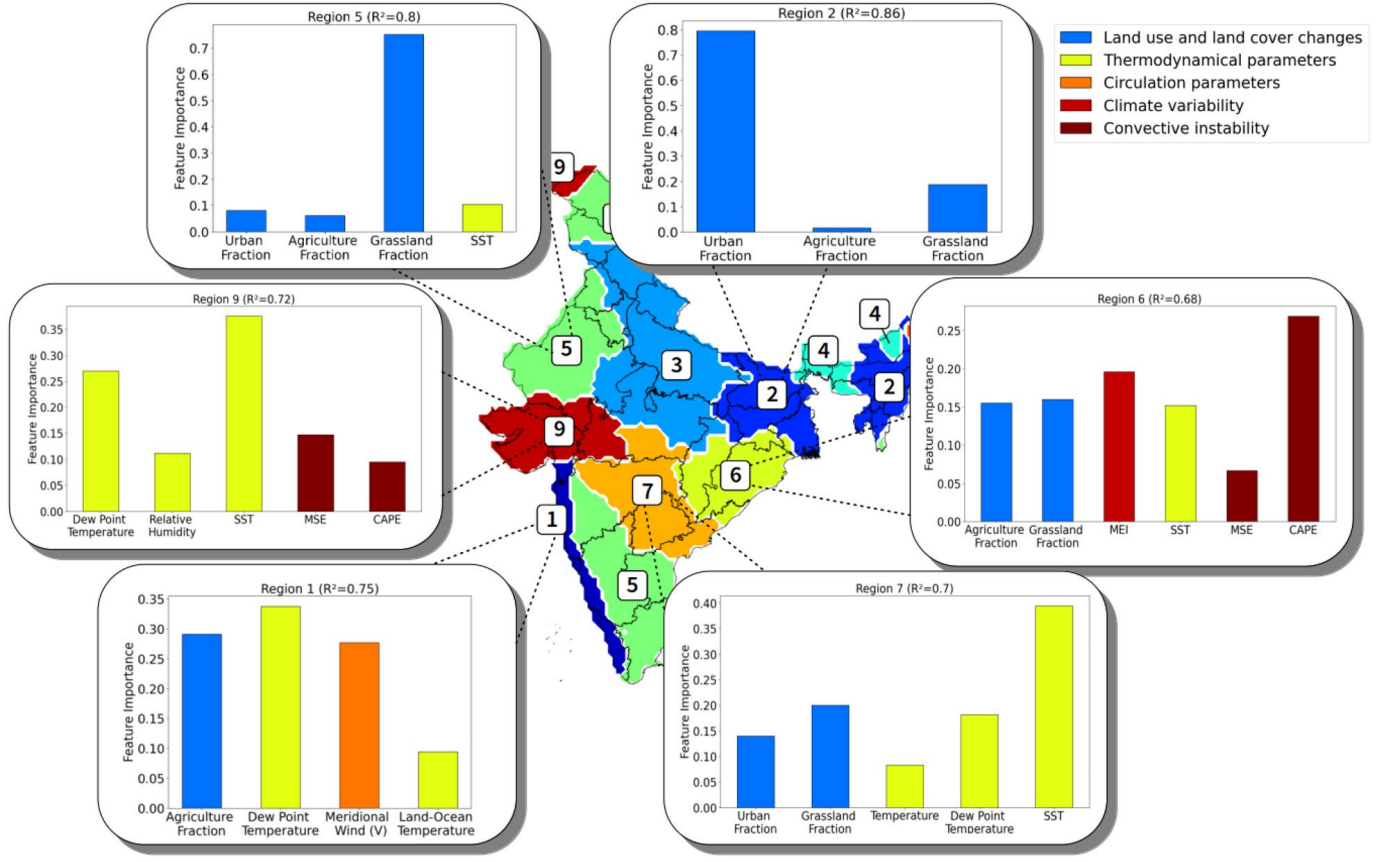
Conditional permutation feature importance for the frequency trends. The most important selected features in the prediction of the frequency of extreme events are displayed in the regions showing a high regression score. The colors of the bars correspond to the five different feature categories (LULCs, thermodynamical, circulation, climate variability and convective instability parameters).

We have included seventeen different input features in the multivariate analysis using a random forest model (Table 1, also Supplementary Materials; Fig. S4), and merged them into five distinct categories in the following discussions for clarity (Fig. 3): (1) LULC changes (composed of four features: agricultural land, grassland, forest, and urban fractions); (2) thermodynamical parameters (temperature, dew point temperature, sea surface temperature or SST, land–ocean temperature gradient, and relative humidity); (3) dynamical circulation parameters (zonal, meridional and total surface wind speed); (4) climate variability indices (El Nino – Southern Oscillation or ENSO and Indian Ocean Dipole or IOD indices); and (5) convective instability parameters (moist static energy or MSE, convective available potential energy or CAPE, and also the number of monsoon depressions forming over the Bay of Bengal). The first category (LULC fractions) differs from other categories in the way that the LULC changes are unarguably both local and anthropogenic features, and their climate responses are also largely regional.

**Table 1:** List of input features of the random forest regression model.

Feature	Category	Computation area
Urban fraction	Land-Use and land cover changes	Regional average
Agricultural fraction	Land-Use and land cover changes	Regional average
Forest fraction	Land-Use and land cover changes	Regional average
Grassland fraction	Land-Use and land cover changes	Regional average
Surface air temperature	Thermodynamical parameters	Regional average
Dew point temperature	Thermodynamical parameters	Regional average
Relative humidity	Thermodynamical parameters	Regional average
Sea surface temperature	Thermodynamical parameters	Arabian Sea
Land–Ocean temperature gradient	Thermodynamical parameters	Arabian Sea and Indian subcontinent
Zonal component of the wind	Circulation parameters	Arabian Sea
Meridional component of the wind	Circulation parameters	Arabian Sea
Wind speed	Circulation parameters	Arabian Sea
MSE	Convective Instability	Arabian Sea
CAPE	Convective Instability	Bay of Bengal
Number of depressions	Convective Instability	Bay of Bengal
ENSO index	Natural climate variability	–
IOD index	Natural climate variability	–

Note that in category (2), (3), and (5), the quantities of included physical parameters can be affected by, besides anthropogenic impacts (e.g., through global warming), global climate processes and feedback involving natural climate system features. The variables in each category were selected based on current knowledge and certain hypotheses of monsoon climatology, as detailed hereafter. Specifically, temperature and humidity are commonly regarded as being correlated to extreme precipitation trends, because the warming of the atmosphere could lead to enhanced moisture availability and hence more intense rainfall^34,35^. However, it was argued that dew point temperature could be a better predictor of extreme events than temperature in the tropics, especially in India^17,18^, hence, we decided to include both features. It has also been suggested that the increase in SST over the Arabian Sea may play a role in the enhancement of extreme events by increasing moisture availability^11,12,24^, while other studies highlighted the more prominent role of the temperature gradient between the ocean and the land on monsoon rainfall^10^. Another key mechanism that has been suggested as a potential cause for the rise in extremes in several previous studies is the monsoon circulation. While some leaned towards the strength^20,36^, others highlighted the direction and variability of the monsoonal flow^12,19^. As most of the moisture over India during the summer monsoon season is advected from the Arabian Sea, in order to evaluate the impact of the monsoon circulation strength on the trends of extremes, we chose to use the zonal and meridional components of the surface wind (U and V respectively), as well as the combined wind speed as input features, all averaged over the Arabian Sea. In addition, some studies have also linked atmospheric instability to precipitation enhancement. For instance, one of our previous studies^37^ argued that the surface MSE is a useful parameter for quantifying the degree of strength of the monsoon convection, while another study^36^ stated that an enhancement in convective instability, measured by calculating the MSE at different pressure levels, could also lead to enhanced precipitations. Furthermore, a recent effort^19^ also identified the increase of convective available potential energy or CAPE over the Bay of Bengal as a potential contributor to the observed intensification of severe storms. The link between monsoon depressions forming over the Bay of Bengal and extreme precipitation events, established on a daily timescale, receives certain acceptance among the tropical meteorology community. Hence, we also included a feature representing the number of depressions per monsoon season, even though it was previously demonstrated that extremes did not show any correlation to the number of depressions at a decadal timescale^12^. Lastly, we would reiterate that we use the long-term climatological evolutions instead of episodic variations of features in the analysis. This better serves the purpose to establish their correlations with observed climatological trend of the monsoonal precipitation extreme events.

### Feature importance for the trends in frequency

For the trends in frequency of extreme events, a very wide variety of factors appear to have played important roles in influencing the observed trends (Fig. 3), as each of the five feature categories is well represented in the list of selected important features. The list of important features, varying substantially from region to region, suggests that different effective predicting factors are behind the rise in the frequency of extremes across these regions. The thermodynamical parameters appear to be the key predicting factors in all the central regions (regions 6, 7 and 9), as well as in the south-western coastal region 1. In particular, the Arabian Sea surface temperature seems to be an important feature of the frequency of extremes in all three central regions, whereas the land–ocean temperature gradient seems to have had a more prominent role in the coastal region 1. It is interesting to note that this region is also the only region where a wind-related feature appears to be correlated to the trend in frequency, whereas the circulation strength is commonly thought to be a good indicator of the monsoon intensity, and thus could potentially drive the trends of extremes. The dew point temperature, which characterizes the quantity of water vapor contained in the atmosphere, also appear to be a top predicting factor in three of the regions. In the eastern coastal region 6, the trend of frequency seems to be dominated by the CAPE, calculated over the Bay of Bengal, as was previously suggested by a previous study^12^. Region 6 is also the only region where the frequency of extremes seems to be impacted by ENSO. Furthermore, the LULC changes are also important predicting factors for the trends of frequency, appearing (at least one of its four components) among the important features in five out of six regions. The above results thus suggest that in causing the frequency increase of extreme events, both local anthropogenic factors and climate variabilities could have played active roles.

### Feature importance for the trends in intensity

**Figure 4:**
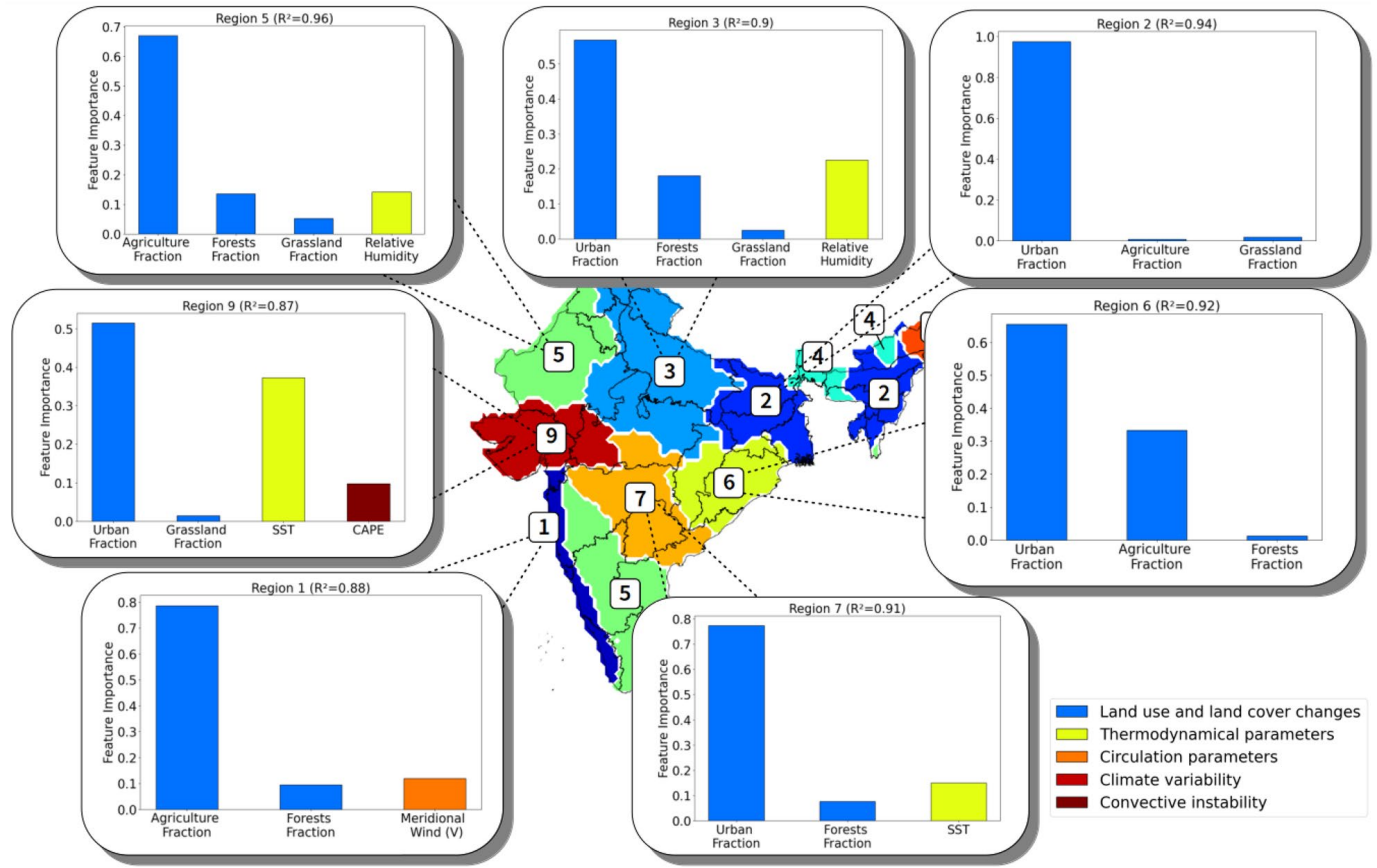
Conditional permutation feature importance for the intensity trends. The most important selected features in the prediction of the intensity of extreme events are displayed in all the regions. The colors of the bars correspond to the five different feature categories (LULCs, thermodynamical, circulation, climate variability and convective instability parameters).

We find that for all regions, the most important features related to the intensity trends of extremes are almost exclusively the LULC changes (Fig. 4). In every study region, we can find at least one of the four LULC fraction features in the leading important feature list, implying that they are crucial for the prediction of extreme events and thus could have played a role in substantially amplifying the rise in intensity of extreme events. As the evolutions of various LULC components are different across regions, their impact on the regional trends could differ. In general, the forest and grassland fractions have decreased over the course of the twentieth century in most studied regions, while agricultural lands and urban areas have increased along with the economic and population growth. Specifically, we find the urban fraction to be the most correlated feature to the intensity trends in five out of seven regions, suggesting that the urbanization may have had an important role in the increase in the intensity of extreme events. In the remaining two regions, the agricultural land fraction appears as the most important feature. Therefore, local anthropogenic factors have largely dominated the prediction of the rising trend of extreme intensity. On the other hand, however, the SST appears as an important feature in two central regions (regions 7 and 9), especially in region 9 where it is ranked in the second position with a relative contribution of almost 40%. The relative humidity also appears in the important feature lists in two of the regions (regions 3 and 5). To a smaller extent, the meridional component of the wind (V) and the CAPE also appear to have impacted the trends in intensity in respectively regions 1 and 9, as in the case of frequency in both regions. Even though the trends in intensity seem to be more correlated to the LULC changes, other physical parameters also appear to have played a role in the intensification of extremes.

### Physical explanation

#### LULC changes

Our machine learning study confirms what has been hypothesized in the earlier trend analysis, that there indeed is a strong marker of the LULC changes in every calculated trend particularly of the intensity of extreme events. LULC changes can impact the regional and global climate through several processes, though their extent and underlying mechanisms may not be well defined to this day^28^. For instance, LULC changes can affect the radiative budget, either by modifying the surface albedo or through effects on the surface latent and sensible heat fluxes, hence atmospheric water vapor or cloud properties. The LULCs would further affect directly or indirectly the local water cycle, by perturbing the evapotranspiration fluxes, or by inducing rainfall modification. In our analysis, we find that the most significantly correlated LULC feature to the long-term trends of extremes are the changes in urban fraction. It is hypothesized that the change in urban areas could modify the rainfall through various mechanisms^38^. Some of them involve land surface heterogeneity feedbacks: convection could increase via thermal perturbations induced by the urban heat island, or the increase in surface roughness^39^. Others are linked to urban anthropogenic emissions, in particular the aerosol indirect effect: urban aerosols can act as cloud condensation nuclei and thus modify cloud microphysics, radiation, and precipitation^40,41^. Previous studies did link the rise in extreme events in some parts of India with urbanization by comparing the pre-urbanization and post-urbanization trends^13,16^, or by analyzing rainfall at different urban locations^15,42^. By exploiting a multivariate analysis using the longest available data, our unique result not only confirms what has been suspected, but also underlines the importance of the other LULC changes. These results are, however, spatially heterogeneous. Some regions, like the Northeastern region 2, seem to display important correlations between the trends of extremes and LULC changes, particularly changes in urban fraction. Note that out of the seven studied regions, region 2 has experienced the most intensive urbanization, where the urban fraction has been leveraged from 0.3% on average in 1901 to 5.5% on average in 2015, or a spectacular + 1670% increase. According to our study, this tremendous increase may have contributed to the rise in the frequency and intensity of extreme events.

#### Thermodynamical parameters

Nevertheless, in certain regions, the markers of urbanization and other LULC changes are not that obvious. For example, in region 9, the region adjacent to the Arabian Sea, there is not any LULC feature appearing in the selected important features of the frequency trend. Instead, SST variations seem to have a dominant role in modulating both frequency and intensity indicators in this region. This could be explained by the fact that a warmer SST would consequently increase the quantity of water vapor over the Arabian Sea through evaporation. Given that the summer monsoonal winds are essentially south-westerly, this additional water vapor would in turn be advected to the Indian subcontinent by the monsoon circulation and enhance moisture supply, thus favoring the occurrence of extreme precipitation events^12^. In region 1, the other coastal region adjacent to the Arabian Sea, the land–ocean temperature gradient appears in the important factor list in addition to the meridional component of the monsoon wind, suggesting that the extreme events in this region are more subject to processes related to the land-sea breeze effect. This finding is also explained by the fact that region 1 is located on the windward side of the mountain range of Western Ghats and receives directly the south-westerly monsoonal winds that bring moist air from the Arabian sea. However, what is surprising is that the wind and land–ocean temperature gradient, commonly believed to be a good indicator of the monsoon strength, do not appear as important predictors for precipitation extremes in the remaining regions, at the considered time scales.

Moreover, it is understood from the Clausius–Clapeyron equation that the atmospheric temperature could be a main driver of the extreme events globally^43^, since a warmer atmosphere can contain a higher quantity of water vapor thus provoke more frequent and more intense rainfall. However, in our analysis, the surface air temperature only appears as an important predictor in region 7 and with very little contribution, whereas the dew point temperature is found to have modulated the trends in frequency in regions 1, 7 and 9, confirming a previous hypothesis stating that it is a better predictor of extremes in tropical regions^17,18^. The reason behind this finding could be that the monsoon precipitation events can actually induce a cooling of the atmosphere due to the high quantity of liquid water evaporation, hence there is no positive correlation between extreme precipitation events and surface air temperature. The positive correlation becomes apparent, however, when looking at the dew point temperature, a direct measure of the absolute humidity of the atmosphere.

#### Global scale climate variability

It is also interesting to note that ENSO seems to be an important predictor for the frequency trend in region 6. While the effects of ENSO on the average monsoon rainfall have been studied and some links have been proposed^44,45^, the relationship between ENSO and extreme precipitation events in India is still not well understood. Future analyses on the correlation between ENSO and extreme precipitation events in this specific region would be required to assess the real impact of this large-scale climatic feature.

#### Convective instability parameters

Finally, the convective instability parameters appear as important predictors of extremes in both west-central (region 9) and east-central coastal region (region 6), especially for the trends of frequency. Particularly, the long-term variation of CAPE calculated over the Bay of Bengal seems to have some impact on the frequency of extreme events in region 6, which is located right next to the Bay of Bengal. By favoring occurrence of strong convective activity, increase in CAPE have been linked to wet spells^19^ and to the intensification of extreme rainfall^46^. Our findings show that it may also have an impact on the long-term trends of frequency of extremes, meaning that a stronger average CAPE during a monsoon season would indicate a higher number of extreme events, in this coastal region.

## Conclusion

The trends of extreme precipitation events defined using two different indicators during the Indian summer monsoon season in the past 120 years have been analyzed. Instead of focusing on a few selected locations, this analysis uniquely covers the entire India, consisting of 9 regions segregated using a clustering method based on precipitation characters. It is found that the majority of India has experienced a statistically significant increase of monsoon precipitation extremes throughout the analyzed 120-year period. Furthermore, the effective predicting factors behind such an increase have also been analyzed using a nonlinear and multivariate machine learning regression, the random forest, based on the best available data of 17 input features describing anthropogenic activities, climate dynamical and physical processes, and variabilities. The results reveal that the man-made land use land cover changes appear to be the most critical features in predicting the observed climatological trends of monsoonal precipitation extremes, implying implicitly that these features might have played an important role in causing the discovered rise of monsoonal precipitation extremes particularly of their intensity. Whereas several climate variability factors including dew point temperature as well as SST, and main monsoonal wind strength, all over the Arabian Sea are also critical to predict the trends of extremes in several regions, especially for their frequency. Nevertheless, certain indicators commonly believed to be drivers for mean monsoonal rainfall strength such as land–ocean thermal contrast, ENSO variation or convective instability are found to be less correlated to the trends of precipitation extremes than expected.

## Methods

### Clustering of regions for analysis

The regions with similar climatological precipitation characters and thus suitable for performing trend analysis were defined by applying Ward’s minimum variance clustering method. Ward’s method is an agglomerative hierarchical clustering method, where each data point is initially considered as a single cluster, then grouped together by calculating the Euclidean distance between them. Here in our case, a data point corresponds to the daily rainfall time series of a single grid cell for the period of 1901–2020, taking only the monsoon days (from June to September included), i.e., the total number of samples is equal to L = 14,640 for each data point. The number of data points N is thus equal to the number of grid cells in the rainfall dataset, i.e., N = 4,954. At the start, each data point is treated as one cluster, so the initial number of clusters is N. Then, a cluster is formed by joining the two closest data points, resulting in N-1 clusters. This step is repeated until one big cluster is formed. The optimal number of clusters is then determined by plotting the dendogram, a figure that represents graphically the distance between data points as well as the distance between clusters, and then choosing the number of clusters that maximizes the inter-cluster distance. We plotted the dendogram using the function of Scikit-learn Python library (https://scikit-learn.org/) applied to a NxL size data matrix.

### Extreme events definitions

For the frequency and intensity indicators, the regional threshold is selected to be equal to the 99th percentile of the total monsoon rainfall distribution in the region, calculated considering only the rainy days of the monsoon seasons for the period 1901–2020. When the daily rainfall value of a grid cell exceeds this threshold, it is counted as an extreme rainfall event. The grid cells are considered individually, therefore for a given day, if the rainfall exceeds the thresholds at two adjacent grid cells, it is counted as two extreme events. To determine the frequency of extreme events in a region, we count the number of extreme rainfall event occurrences in the region for each monsoon season. To determine the intensity of extreme events, we calculate the average rainfall rate of the previously defined extreme rainfall events for each monsoon season.

### Rainfall extreme trend derivation

To calculate the trends of rainfall extreme events, we performed a Mann–Kendall trend test, a non-parametric test which purpose is to statistically assess if there is an upward or downward trend in a time series. The trend tests have all been performed on the 5% significance level. We then used Theil-Sen estimator to calculate the slope of the established trends.

### RSD t-test

The detailed method can be found in Zuo et al. (2019)^29^. We chose a trend turning timescale T of thirty years, since we wanted to assess the different multi-decadal trends. Let y1 and y2 be the first and last year of the times series. For each year y in [y1 + T, y2-T], we calculate the two slopes of the sub-time series of extremes of length T prior and post y. We then perform a statistical test on the slope difference. If the slope prior y is significantly different than the slope post y, it means that a potential trend turning may occur at year y. The statistical test of slope difference is performed with a t-distribution statistic.

### Random forest regression

We determined the main driving factors of the different trends using the random forest regression^30^, a non-linear supervised ensemble machine learning algorithm that uses multiple decision trees to fit targeted output (e.g., extreme events trends in this study) with selected input data or features (see “Features in random forest regression” section). It operates by constructing a multitude of decision trees at training time and outputting the mean predictions of the individual trees. A decision tree is constructed using two kinds of elements: nodes and branches. The algorithm recursively breaks down the initial dataset into smaller and smaller subsets by evaluating each feature and using at each node the feature that best splits the data (i.e., that returns the highest reduction of a particular variance metric), while in the meantime the decision tree is incrementally developed.

This construction process can be summarized with these steps:

- Step 1: The variance of the target is calculated (here, the target is the extreme events values).
- Step 2: The dataset is split using the different features. The resulting variance for each branch is calculated and subtracted from the variance before the split to obtain the variance reduction.
- Step 3: The feature with the largest variance reduction is chosen for the decision node.
- Step 4: The dataset is divided based on the values of the selected feature. This process is recursively repeated until all data is processed based on chosen thresholds.

The prediction of a new sample is simply calculated using the path created by the decision tree and averaging the values of the samples in the final node (also called leaf node).

### Feature importance and feature selection

We first used the cforest function of the R Party package library to perform random forest regressions of observed extreme trends for each region using various input features. Up on the success of regression, we then applied the conditional permutation feature importance functionality^33^ to determine the importance ranking of each one of the 17 input features, while minimizing the impact of the multi-collinearity. To perform the feature selection, we calculated the average importance of the features, and removed the features that showed an importance inferior to the mean importance. This finally yielded between 3 to 6 important selected features in the different regions, which are displayed in Figs. 3 and 4. To identify the driving factors in predicting the long-term trends, we used the 10-year moving averages of the input data and the output measures of extreme events. This manages to smooth out the noise and inter-annual variability while keeping the longer-term variations.

### Features in random forest regression

We have selected seventeen features in random forest regression and feature important analysis (Table 1). For local anthropogenic activities, we included four land use and land cover features: agricultural land, grassland, forest, and urban fractions. In addition, we have also included certain climate features: surface air temperature, dew point temperature, relative humidity, sea surface temperature or SST, land–ocean temperature gradient, zonal as well as meridional components of the surface wind over the Arabian Sea, and associated combined wind speed, El Nino – Southern Oscillation or ENSO and Indian Ocean Dipole or IOD indices, moist static energy or MSE, and convective available potential energy or CAPE. We also included the number of depressions forming over the Bay of Bengal per year.

Some input features are calculated within each region, such that the input trends are region dependent. This is the case for the LULC changes, surface temperature, dew point temperature, and relative humidity. For these features, their annual values were derived from monsoon seasonal and regional means. Other features (U and V, wind speed, SST and MSE) have been calculated over the Arabian Sea, as the moist monsoon winds that provokes precipitation are essentially south-westerly, while the CAPE has been derived over the Bay of Bengal, following considerations from previous studies. All these parameters have been calculated at the ocean surface. The land–ocean temperature gradient has been calculated by taking the surface air temperature difference between the Arabian Sea and the Indian subcontinent. Finally, we also tested the influence of large-scale climate variabilities including the El-Nino Southern Oscillation (ENSO) and the Indian Ocean Dipole (IOD), by using the Extended Multivariate ENSO Index (MEI) v2 and the Dipole Mode Index (DMI) averaged over the summer monsoon seasons as input features. Before fitting the model, each feature is normalized to a range of [−1, 1]. The list of features is detailed in Table 1.

### Model accuracy

To evaluate the accuracy of the random forest model, we used the common train/test split method, which consists in fitting the model with a random subset of the data, and then testing the accuracy with the remaining testing data. This process is repeated fifty times to ensure that the random subsets cover the whole range of our initial dataset, the final score being the averaged value of these fifty scores. Here, 70% of the input data is chosen randomly to train the model, and the regression score corresponds to the coefficient of determination R^2^ of the prediction, defined as:

$$R^{2}=1-\frac{u}{v}$$

where $u=\sum \left({y}_{test}-{y}_{pred}\right)^{2}$ is the residual sum of squares, and $v=\sum \left({y}_{test}-{{\overline{y}}_{{test}}}\right)^{2}$ the total sum of squares, y_test_ and y_pred_ being respectively the value of the testing data and the value predicted by the model.

The best possible score is 1 and corresponds to a model that predicts exactly the right value. The score can also be negative if it fails to deliver any information on the data.

## Supplementary Information

Supplementary Figures.

## Data Availability

We used the daily rainfall gridded dataset at 0.25° × 0.25° resolution delivered by the Indian Meteorological Department or IMD^47^ to derive the precipitation extremes. For the physical parameters including the wind components, temperature and humidities, we used the twentieth Century Reanalysis Dataset version 3^48^. For the ENSO index, we used the Extended Multivariate ENSO Index or MEI.ext^49^, it can be obtained from https://psl.noaa.gov/enso/mei.ext/#data. For the IOD index, we used the Dipole Mode Index (DMI) calculated by NOAA ESRL Physical Sciences Laboratory, accessible from https://psl.noaa.gov/gcos_wgsp/Timeseries/DMI/. For the LULC changes, we used data reconstructed by combining high-resolution remote sensing datasets and inventory archives^27^. For the number of monsoon depressions forming over the Bay of Bengal, we used the cyclone eAtlas data delivered by the IMD (http://www.imdchennai.gov.in/cyclone_eatlas.htm). All the other data are available from the corresponding authors on reasonable request.
