# Supplementary material for: The rise of Indian summer monsoon precipitation extremes and its correlation with long-term changes of climate and anthropogenic factors
Source: Sci Rep. 2022 Jul 14;12:11985. doi: 10.1038/s41598-022-16240-0 (PMC9283463; doi:10.1038/s41598-022-16240-0)
Supplement: Supplementary file 1 — Supplementary Figures. [file 41598_2022_16240_MOESM1_ESM.pdf]

The rise of Indian summer monsoon precipitation extremes and its correlation with long-term changes of climate and anthropogenic factors

Renaud Falga and Chien Wang*

Laboratoire d'Aérologie, University of Toulouse III – Paul Sabatier, Toulouse, France

* email: chien.wang@aero.obs-mip.fr

Supplementary Material

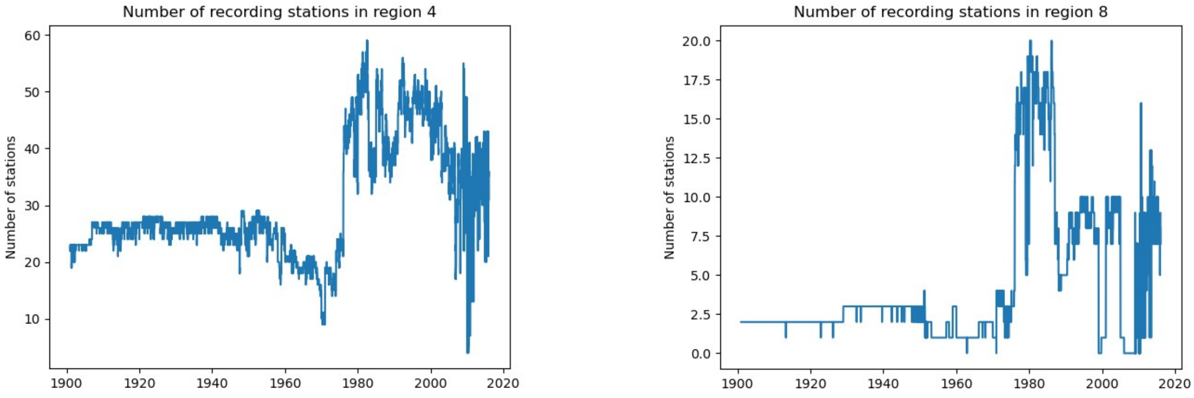

Fig. S1 | Number of recording stations in regions 4 and 8. The plots show the total number of recording stations in region 4 (left panel) and region 8 (right panel). We see a sudden increase in the number of recording stations in the 1970's in both regions, which may have had some impact on the resulting calculated extremes. In region 8, there were almost no recording stations in the region until this time. We don't witness such a sudden change in any other region.

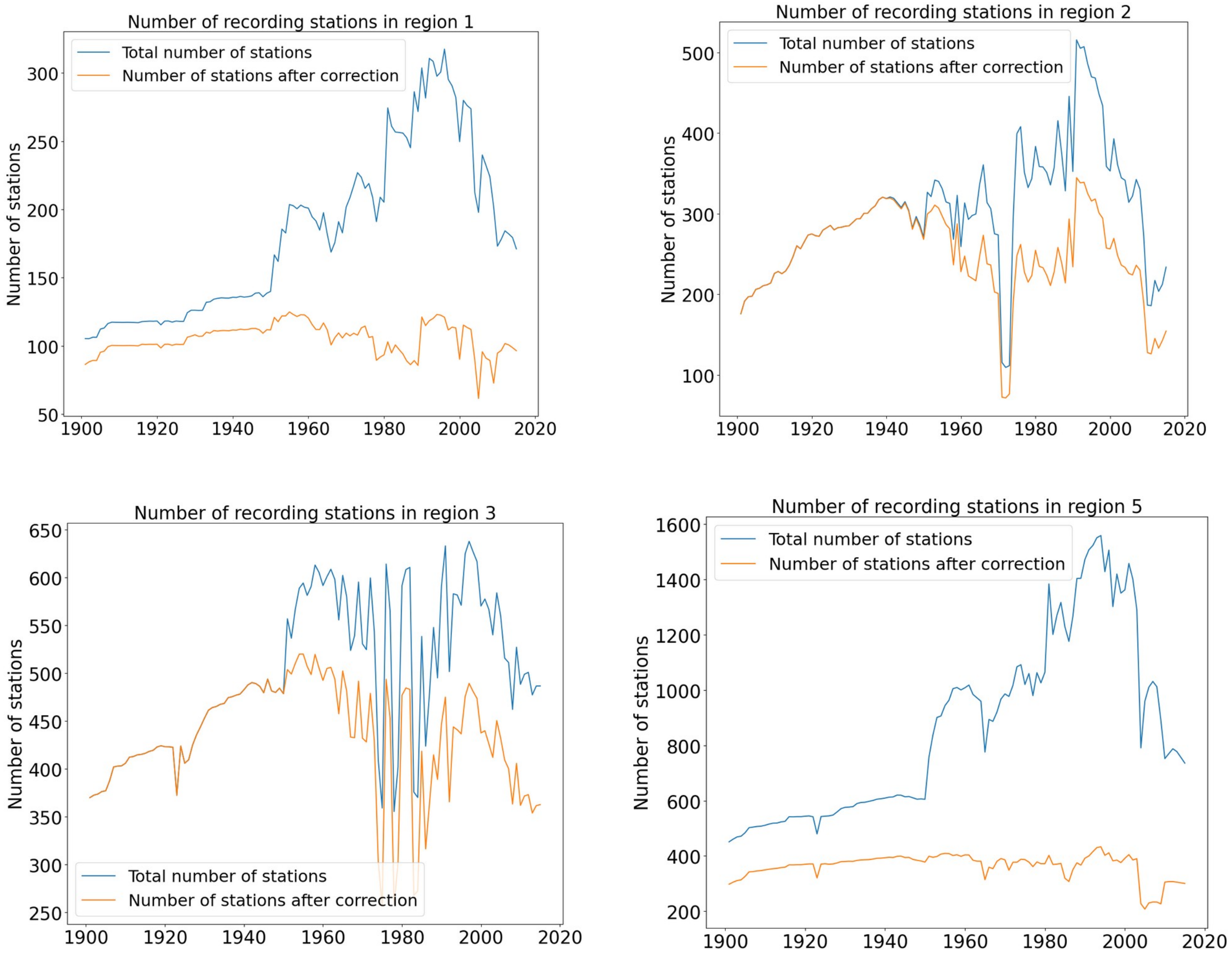

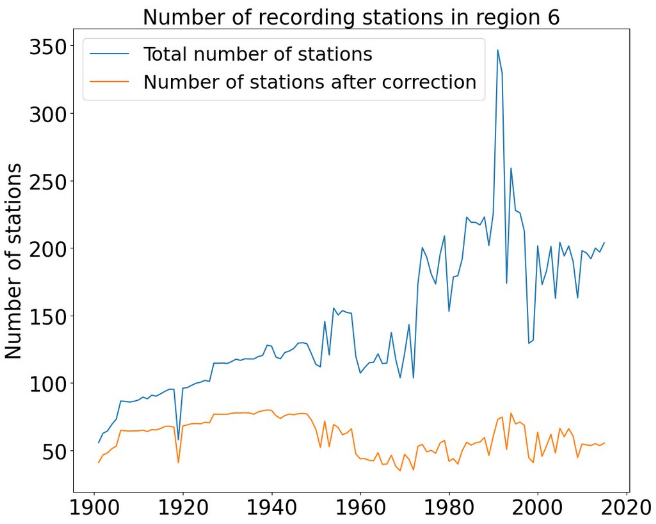
Number of recording stations in region 6
Total number of stations
Number of stations after correction
Number of stations

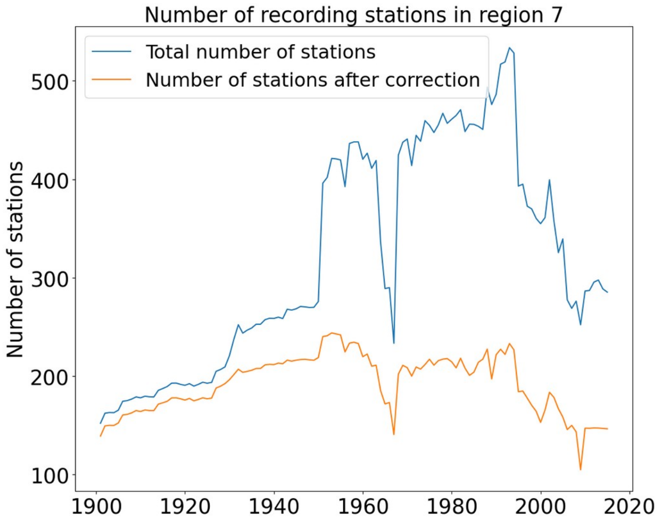
Number of recording stations in region 7
Total number of stations
Number of stations after correction
Number of stations

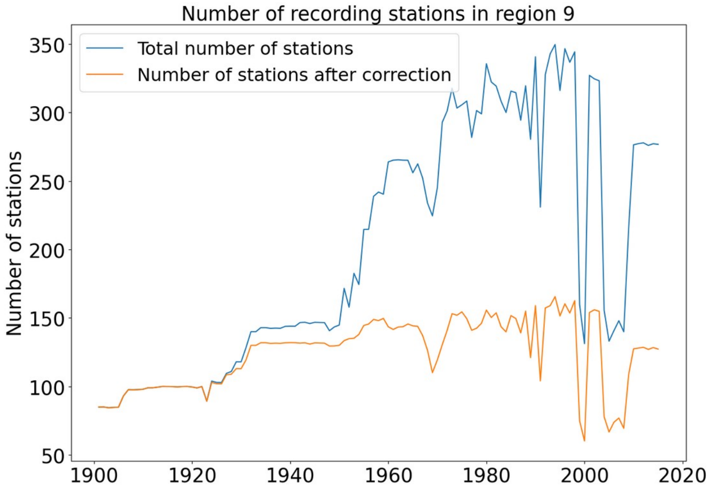
Number of recording stations in region 9
Total number of stations
Number of stations after correction
Number of stations

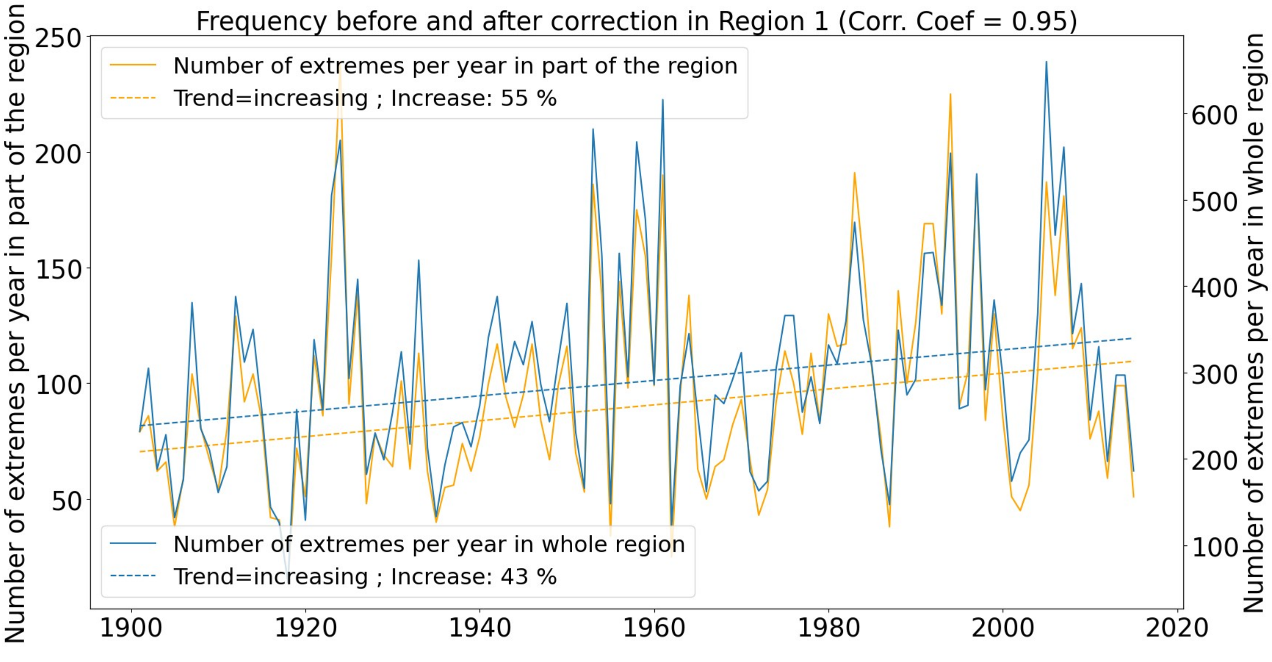
Frequency before and after correction in Region 1 (Corr. Coef = 0.95)
Number of extremes per year in part of the region
Trend=increasing ; Increase: 55 %
Number of extremes per year in whole region
Trend=increasing ; Increase: 43 %
Number of extremes per year in part of the region
Number of extremes per year in whole region

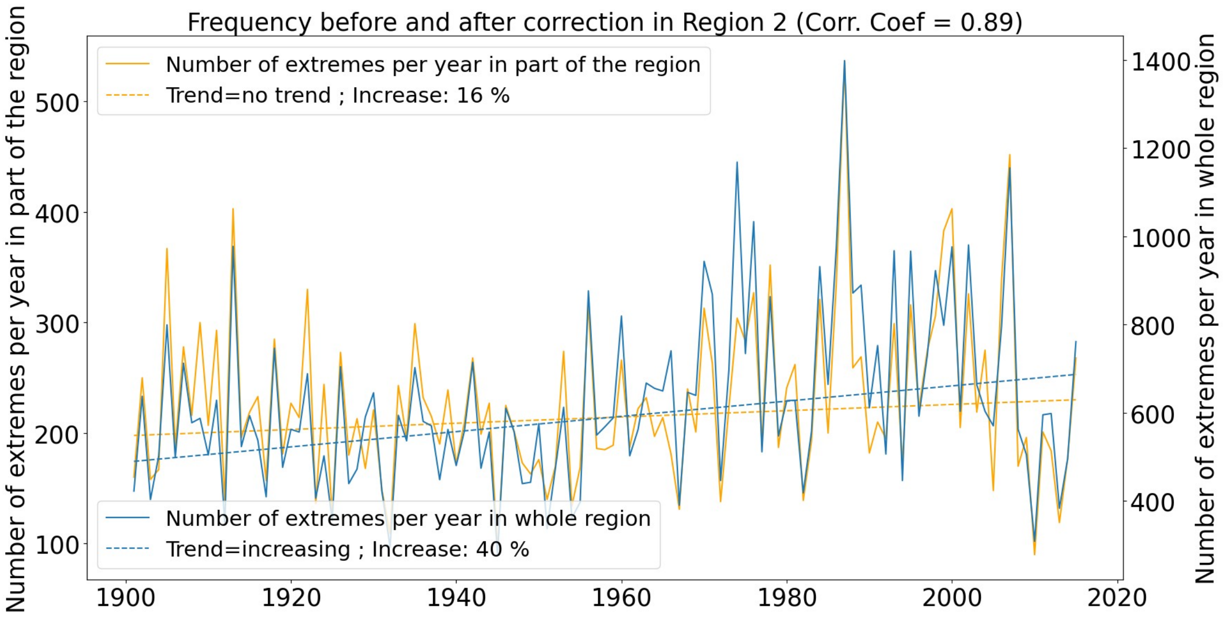
Frequency before and after correction in Region 2 (Corr. Coef = 0.89)
Number of extremes per year in part of the region
Trend=no trend ; Increase: 16 %
Number of extremes per year in whole region
Trend=increasing ; Increase: 40 %
Number of extremes per year in part of the region
Number of extremes per year in whole region
100
200
300
400
500
400
600
800
1000
1200
1400
1900
1920
1940
1960
1980
2000
2020

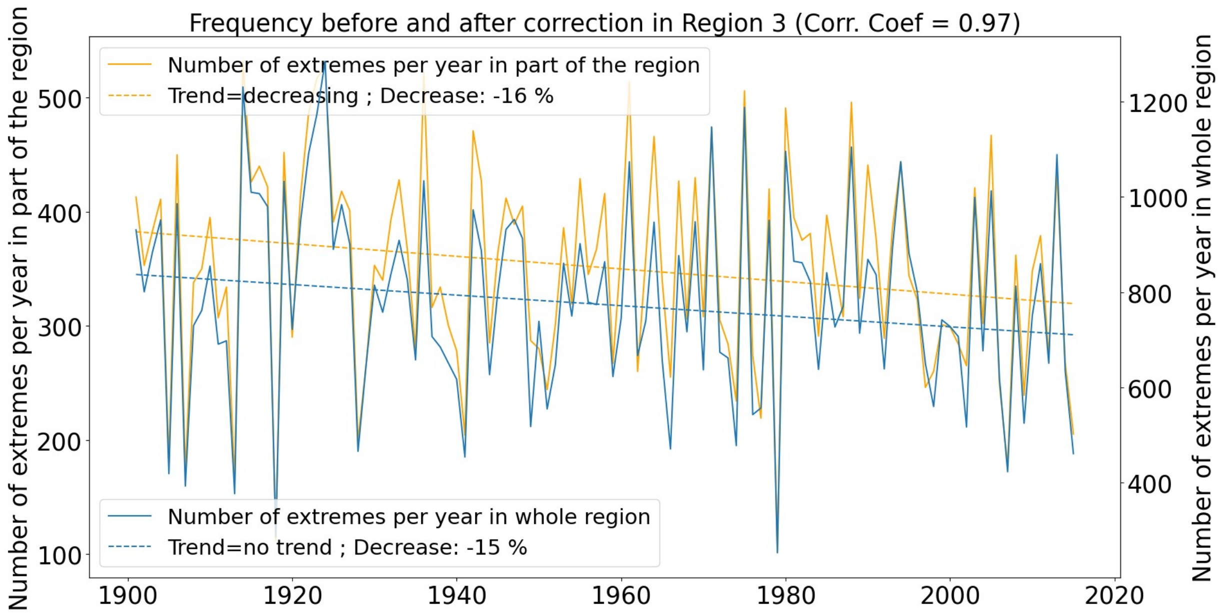
Frequency before and after correction in Region 3 (Corr. Coef = 0.97)
Number of extremes per year in part of the region
Trend=decreasing ; Decrease: -16 %
Number of extremes per year in whole region
Trend=no trend ; Decrease: -15 %
Number of extremes per year in part of the region
Number of extremes per year in whole region
100
200
300
400
500
400
600
800
1000
1200
1900
1920
1940
1960
1980
2000
2020

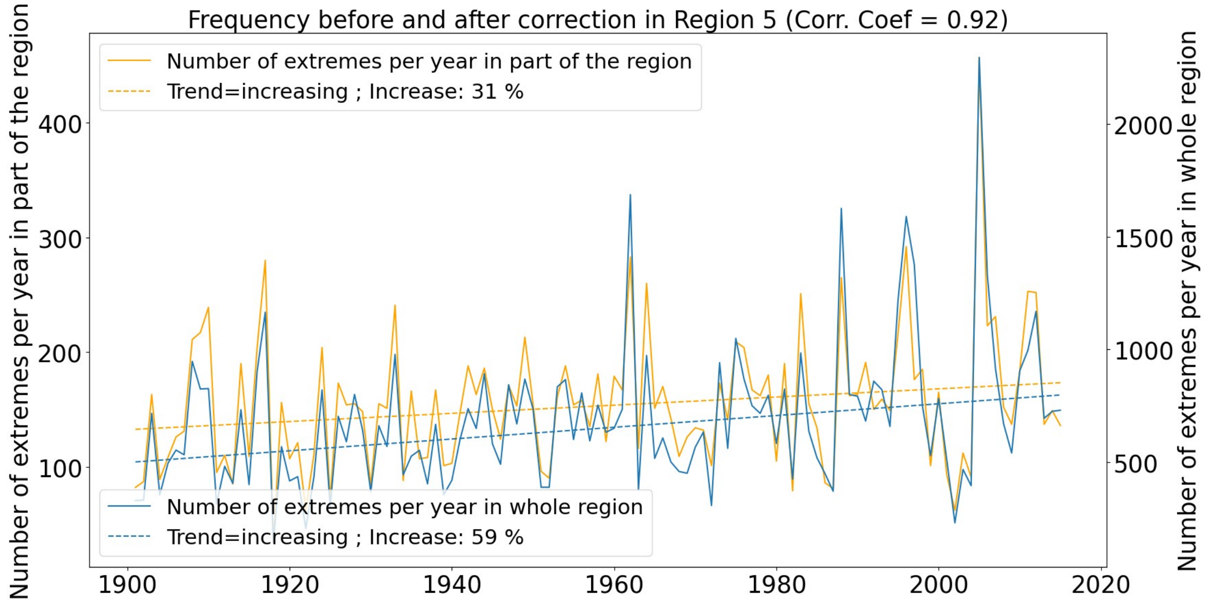
Frequency before and after correction in Region 5 (Corr. Coef = 0.92)
Number of extremes per year in part of the region
Trend=increasing ; Increase: 31 %
Number of extremes per year in whole region
Trend=increasing ; Increase: 59 %
Number of extremes per year in part of the region
Number of extremes per year in whole region
100
200
300
400
500
1000
1500
2000
1900
1920
1940
1960
1980
2000
2020

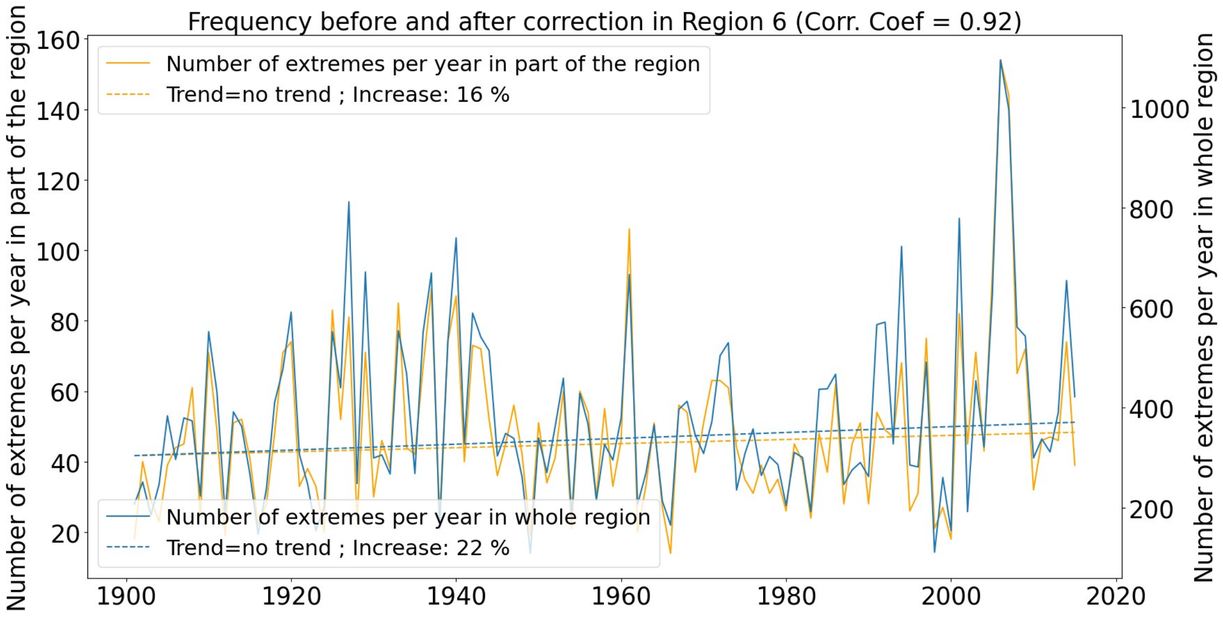
Frequency before and after correction in Region 6 (Corr. Coef = 0.92)
Number of extremes per year in part of the region
Trend=no trend ; Increase: 16 %
Number of extremes per year in whole region
Trend=no trend ; Increase: 22 %
Number of extremes per year in part of the region
Number of extremes per year in whole region
20
40
60
80
100
120
140
160
200
400
600
800
1000
1900
1920
1940
1960
1980
2000
2020

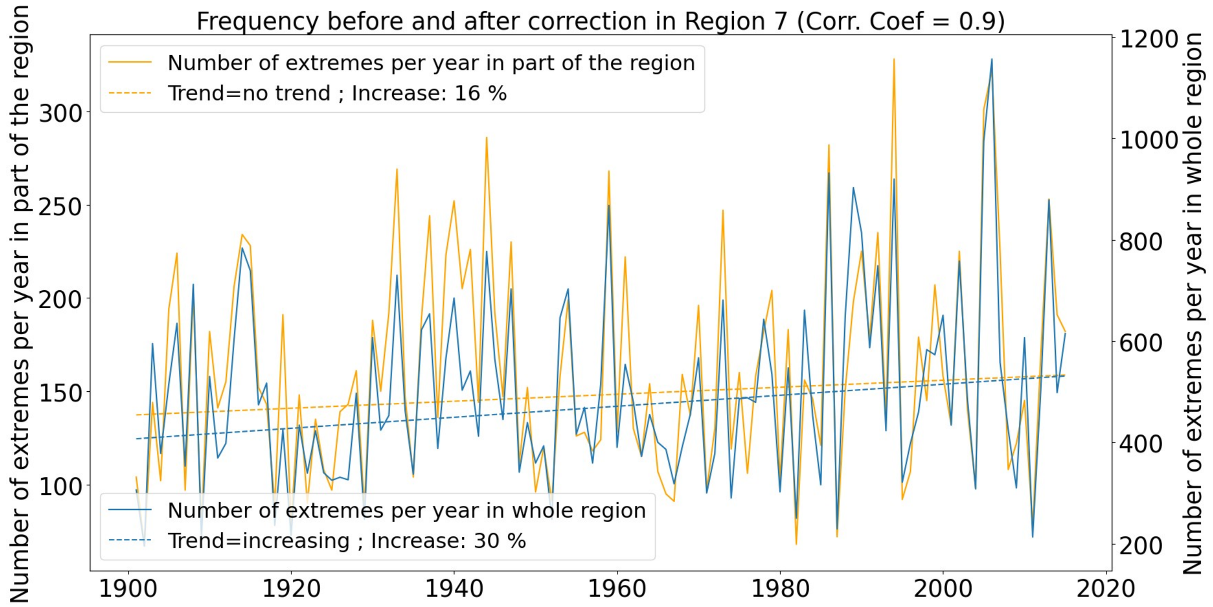
Frequency before and after correction in Region 7 (Corr. Coef = 0.9)
Number of extremes per year in part of the region
Trend=no trend ; Increase: 16 %
Number of extremes per year in whole region
Trend=increasing ; Increase: 30 %
Number of extremes per year in part of the region
Number of extremes per year in whole region
100
150
200
250
300
200
400
600
800
1000
1200
1900
1920
1940
1960
1980
2000
2020

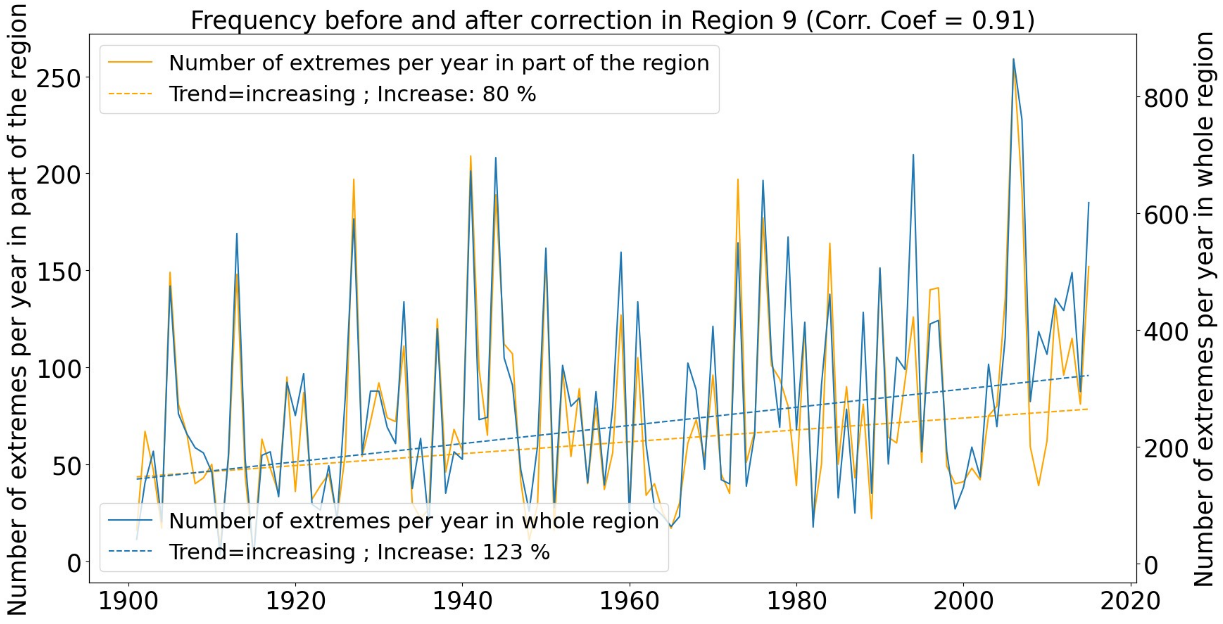
Frequency before and after correction in Region 9 (Corr. Coef = 0.91)
Number of extremes per year in part of the region
Trend=increasing ; Increase: 80 %
Number of extremes per year in whole region
Trend=increasing ; Increase: 123 %
Number of extremes per year in part of the region
Number of extremes per year in whole region
0
50
100
150
200
250
0
200
400
600
800
1900
1920
1940
1960
1980
2000
2020

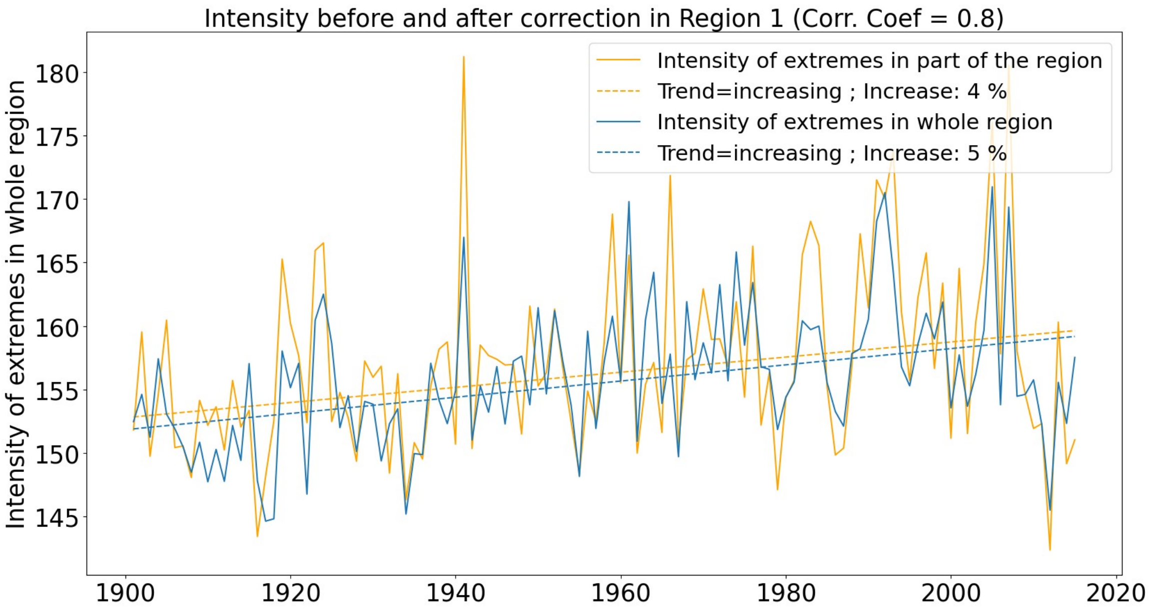
Intensity before and after correction in Region 1 (Corr. Coef = 0.8)
Intensity of extremes in part of the region
Trend=increasing ; Increase: 4 %
Intensity of extremes in whole region
Trend=increasing ; Increase: 5 %
Intensity of extremes in whole region
180
175
170
165
160
155
150
145
1900
1920
1940
1960
1980
2000
2020

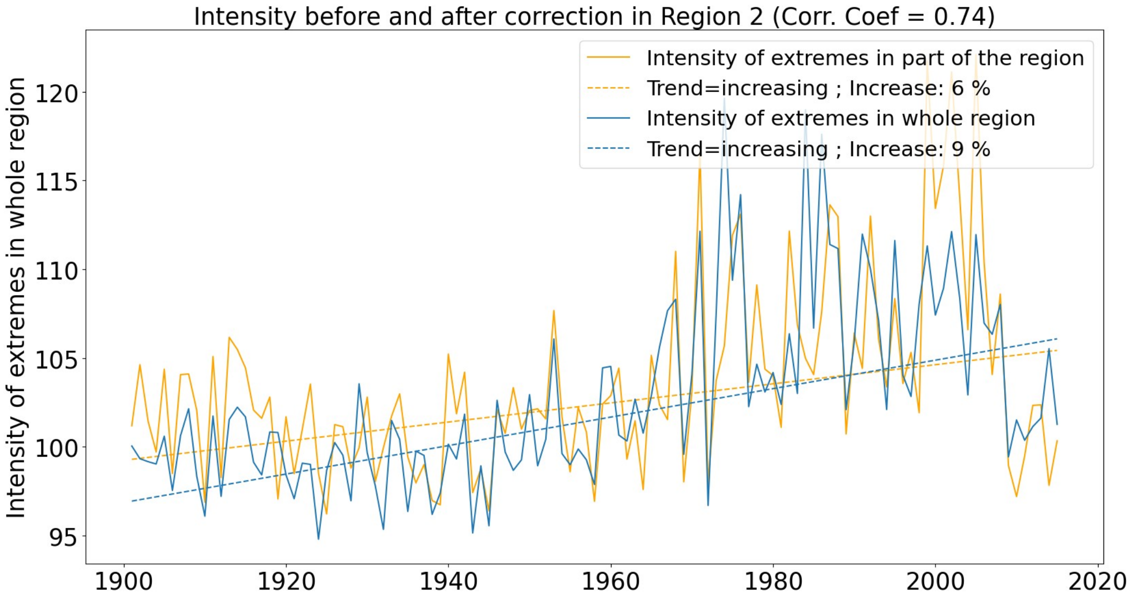
Intensity before and after correction in Region 2 (Corr. Coef = 0.74)
Intensity of extremes in part of the region
Trend=increasing ; Increase: 6 %
Intensity of extremes in whole region
Trend=increasing ; Increase: 9 %
Intensity of extremes in whole region
120
115
110
105
100
95
1900
1920
1940
1960
1980
2000
2020

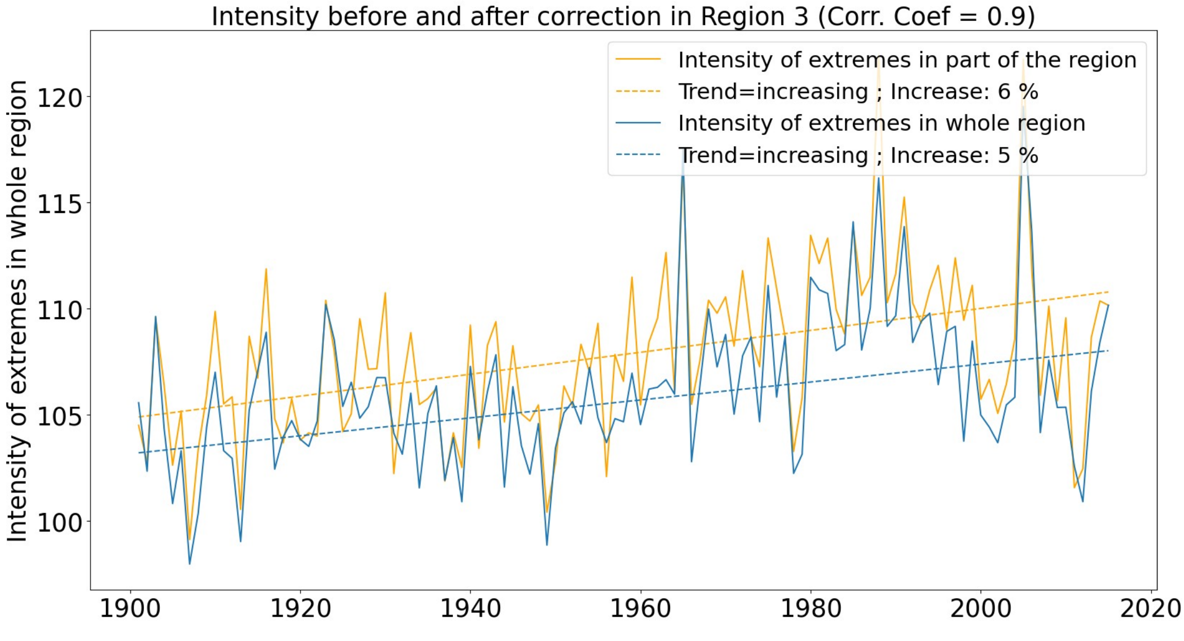
Intensity before and after correction in Region 3 (Corr. Coef = 0.9)
Intensity of extremes in part of the region
Trend=increasing ; Increase: 6 %
Intensity of extremes in whole region
Trend=increasing ; Increase: 5 %
Intensity of extremes in whole region
120
115
110
105
100
1900
1920
1940
1960
1980
2000
2020

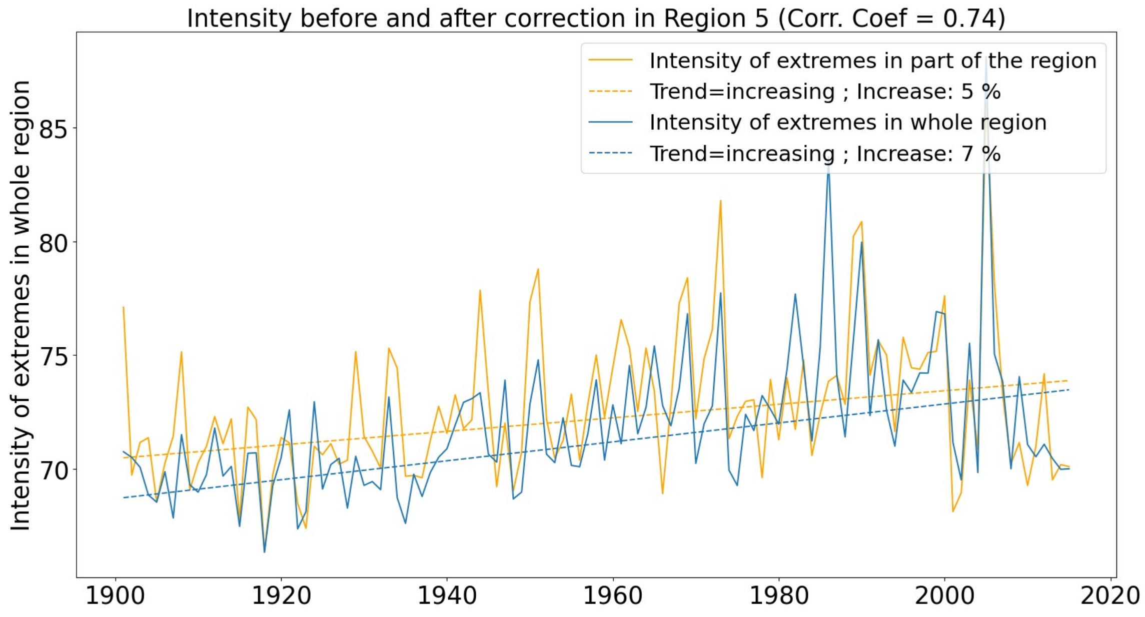
Intensity before and after correction in Region 5 (Corr. Coef = 0.74)
Intensity of extremes in part of the region
Trend=increasing ; Increase: 5 %
Intensity of extremes in whole region
Trend=increasing ; Increase: 7 %
Intensity of extremes in whole region
70
75
80
85
1900
1920
1940
1960
1980
2000
2020

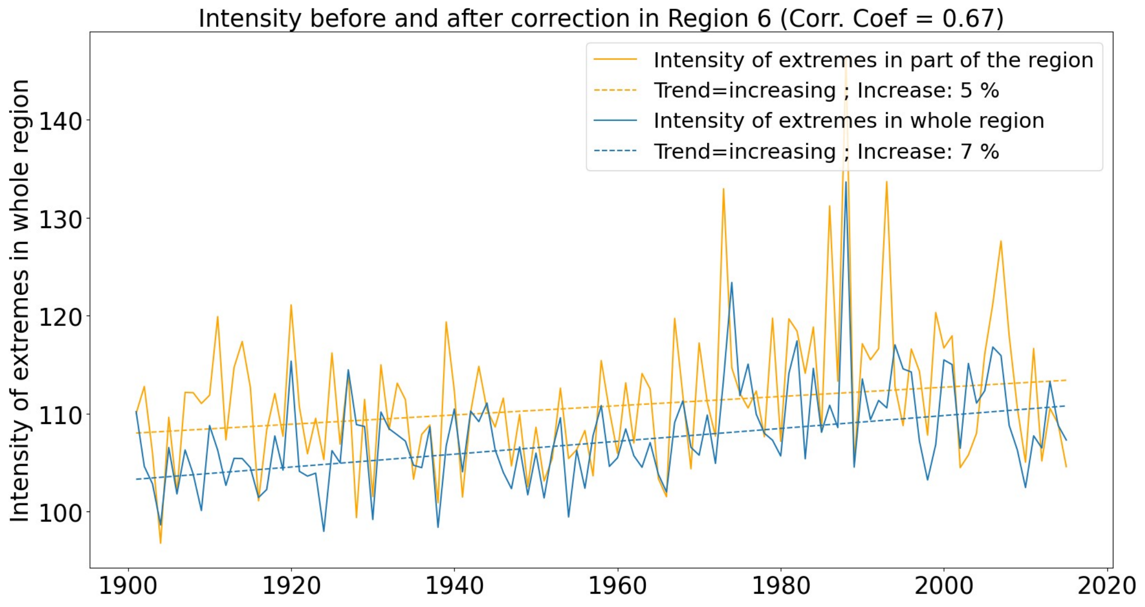
Intensity before and after correction in Region 6 (Corr. Coef = 0.67)
Intensity of extremes in part of the region
Trend=increasing ; Increase: 5 %
Intensity of extremes in whole region
Trend=increasing ; Increase: 7 %
Intensity of extremes in whole region
100
110
120
130
140
1900
1920
1940
1960
1980
2000
2020

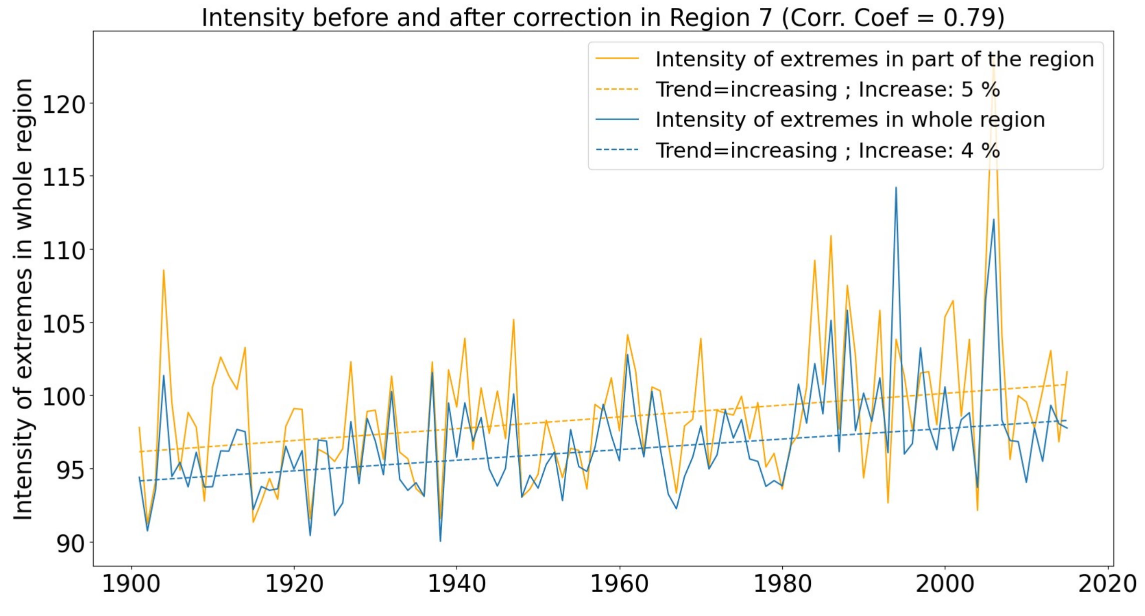
Intensity before and after correction in Region 7 (Corr. Coef = 0.79)
Intensity of extremes in part of the region
Trend=increasing ; Increase: 5 %
Intensity of extremes in whole region
Trend=increasing ; Increase: 4 %
Intensity of extremes in whole region
90
95
100
105
110
115
120
1900
1920
1940
1960
1980
2000
2020

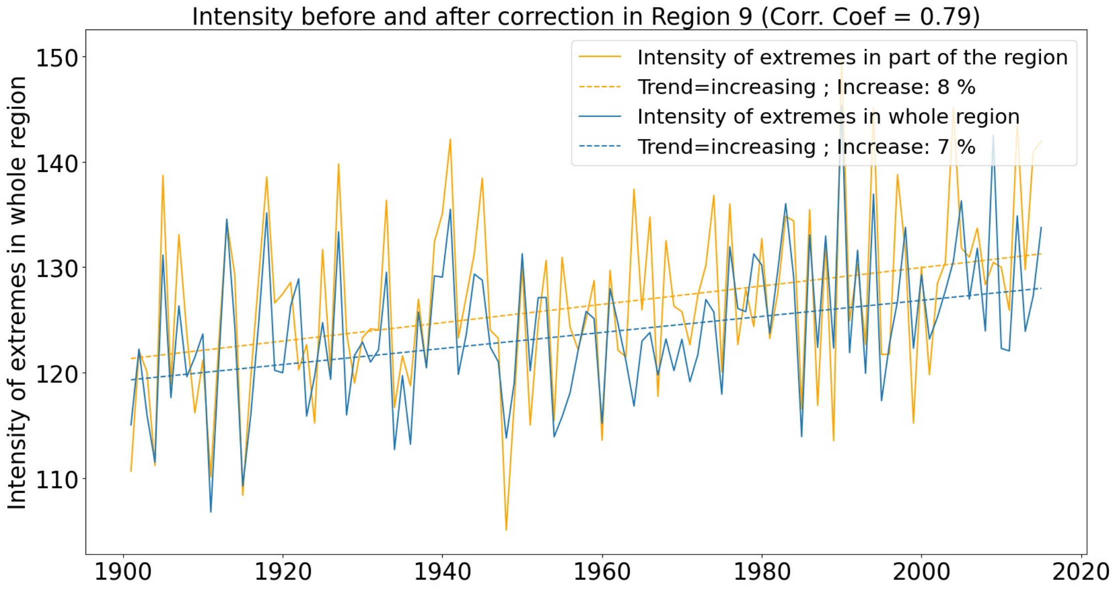

Fig. S2 | Trend comparison before and after correction on the number of observational stations. The number of observational stations for the past 120 years has been derived in each region, as well as the number of observational stations when keeping only the grid cells where the number of stations is stable throughout the century (a). The trends of extreme events before and after correction (i.e., using every grid cell in the region versus using only the ones where the number of stations is stable) have been compared (b) in order to assess the impact of the number of recording stations on the trends. The trends before and after correction are found to be similar.

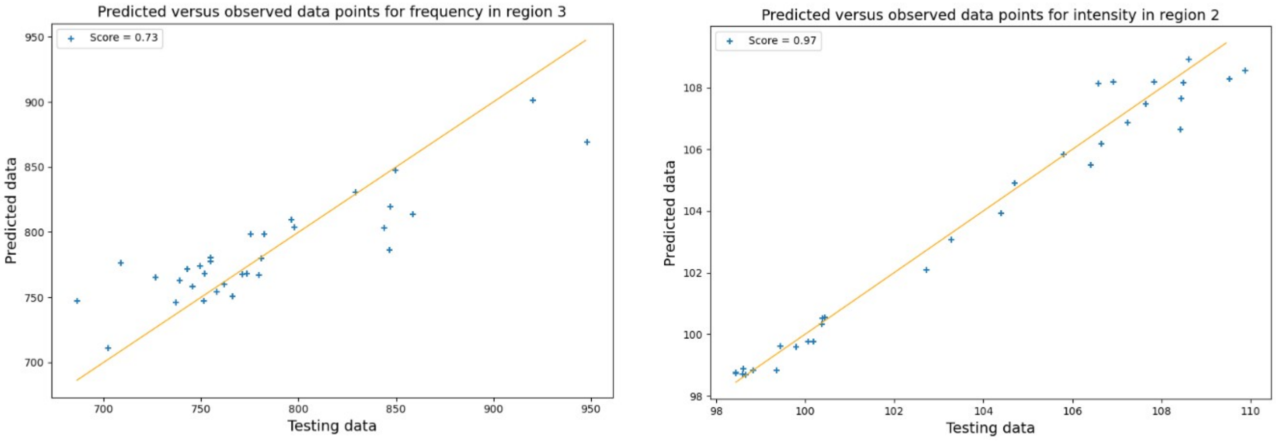

Fig. S3 | Model prediction versus observation. Scatter plots of extreme events predicted by the model (y axis) and observations (x axis) for the lowest (left panel) and the highest (right panel) predicting scores. The left panel values of extreme events are given in number of occurrence per year, while the right panel values are given in mm/day. The scatter plots show the results for a single testing subset of the data. The closer the scatter points are to the y=x axis (shown in orange), the more accurate is the prediction.

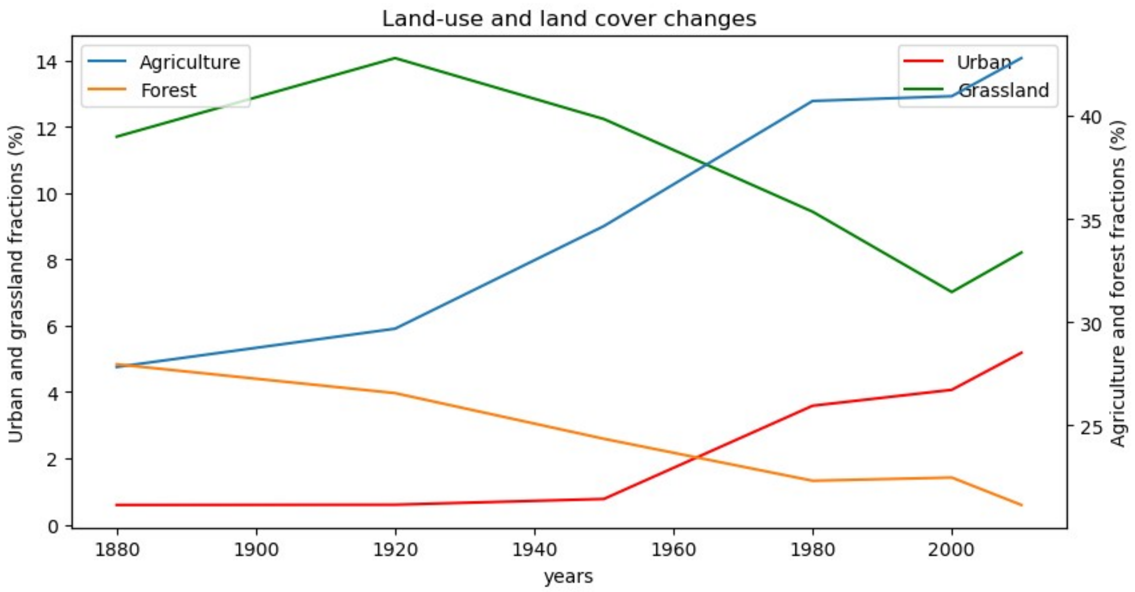
Land-use and land cover changes
Agriculture
Forest
Urban
Grassland
Urban and grassland fractions (%)
Agriculture and forest fractions (%)
years
0
2
4
6
8
10
12
14
25
30
35
40
1880
1900
1920
1940
1960
1980
2000

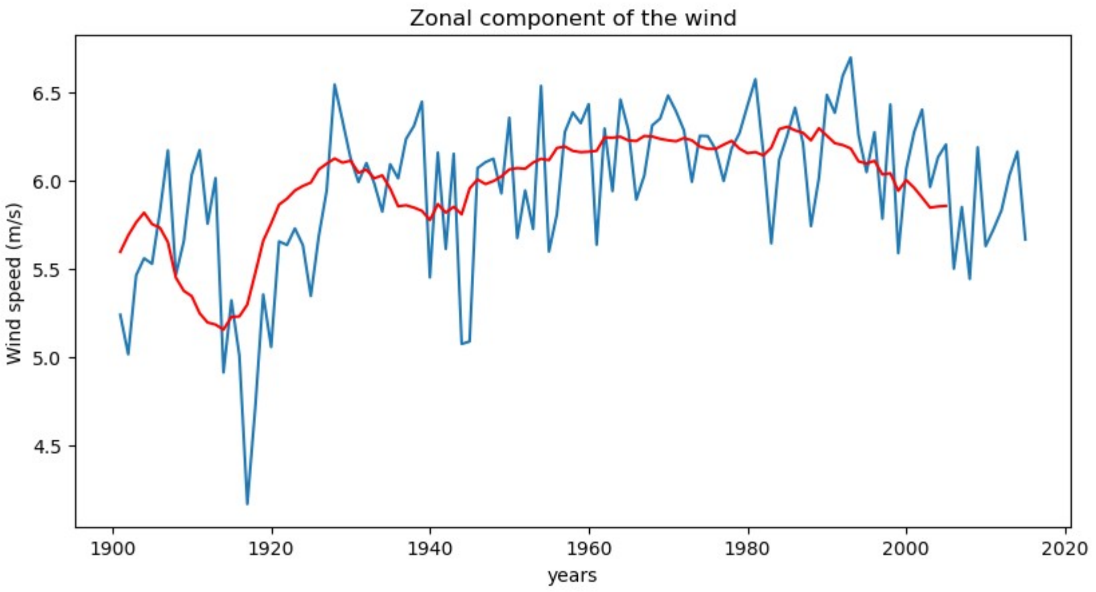
Zonal component of the wind
Wind speed (m/s)
years
4.5
5.0
5.5
6.0
6.5
1900
1920
1940
1960
1980
2000
2020

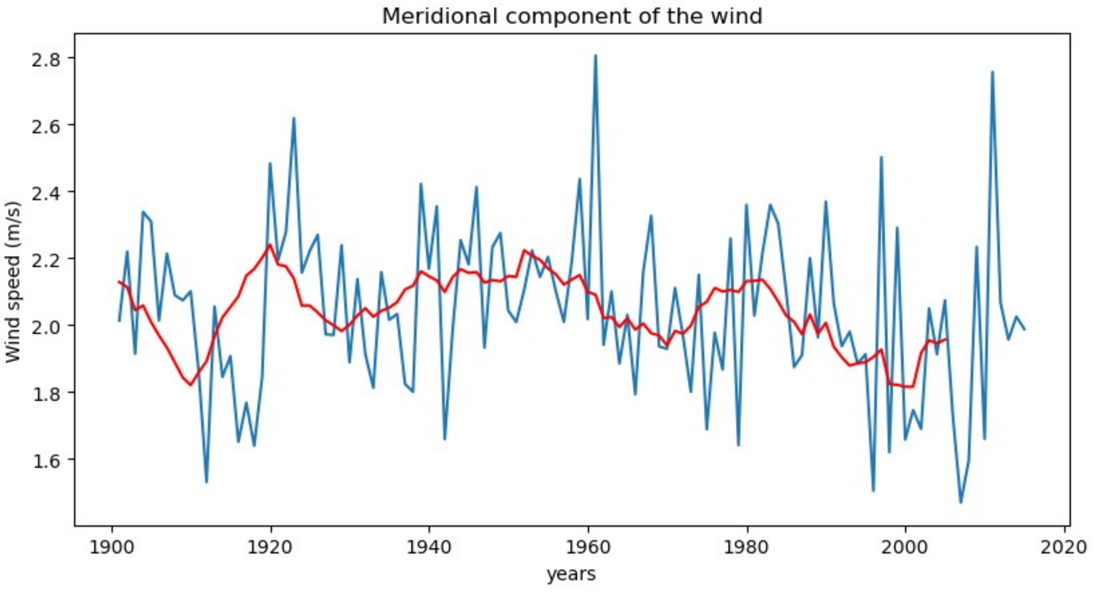
Meridional component of the wind
Wind speed (m/s)
years
1.6
1.8
2.0
2.2
2.4
2.6
2.8
1900
1920
1940
1960
1980
2000
2020

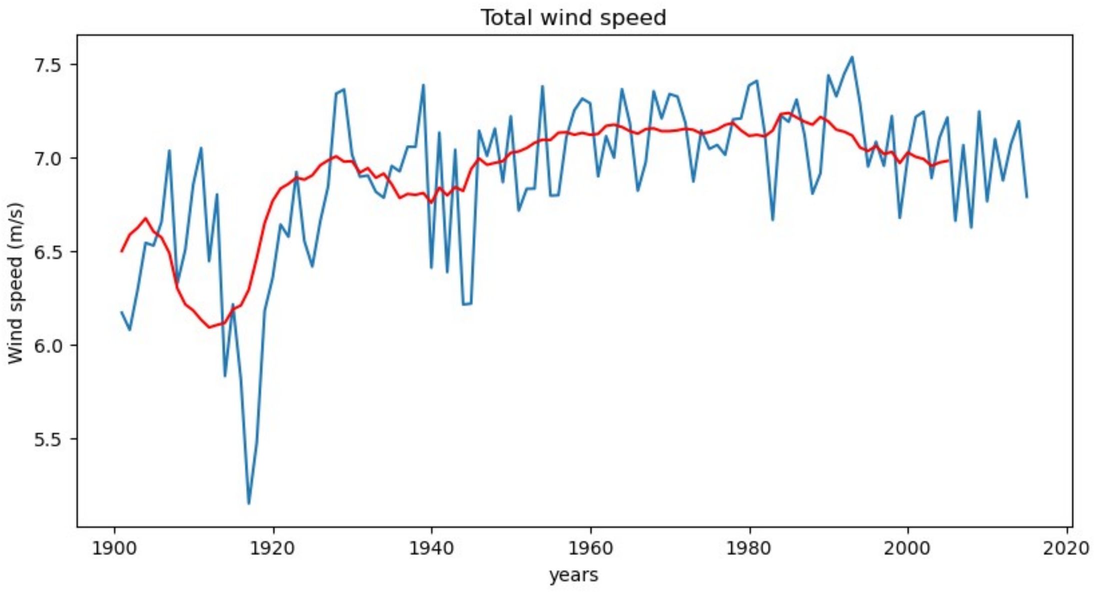
Total wind speed
Wind speed (m/s)
7.5
7.0
6.5
6.0
5.5
1900
1920
1940
1960
1980
2000
2020
years

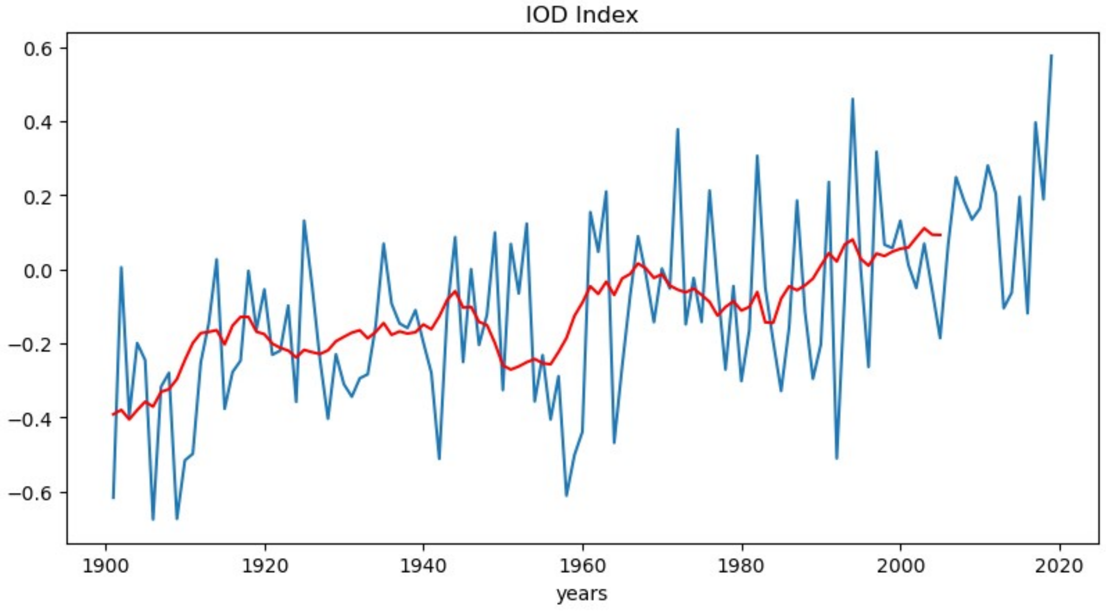
IOD Index
0.6
0.4
0.2
0.0
−0.2
−0.4
−0.6
1900
1920
1940
1960
1980
2000
2020
years

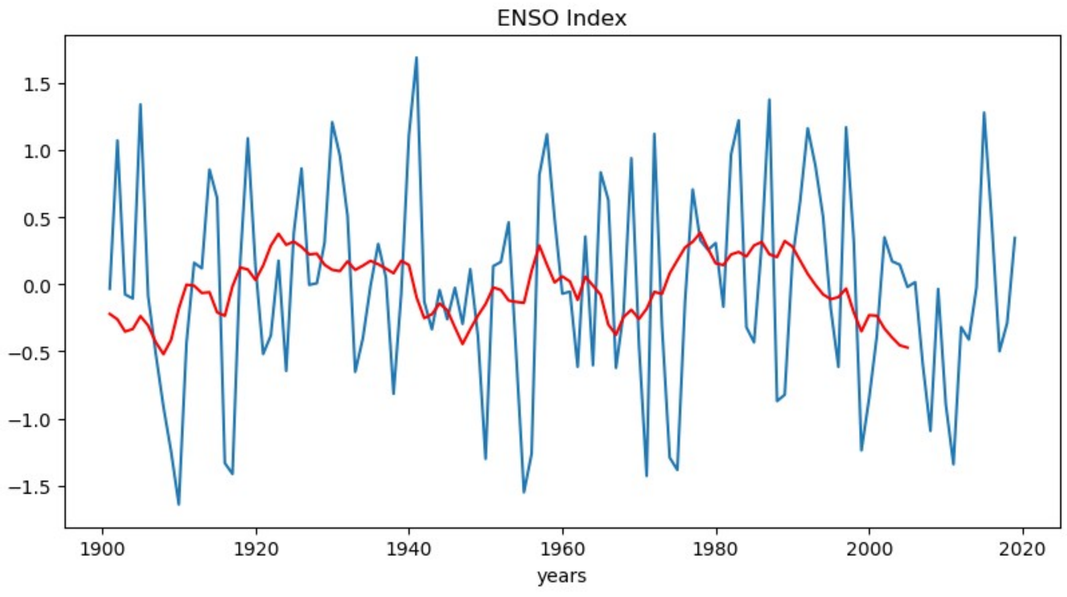
ENSO Index
1.5
1.0
0.5
0.0
−0.5
−1.0
−1.5
1900
1920
1940
1960
1980
2000
2020
years

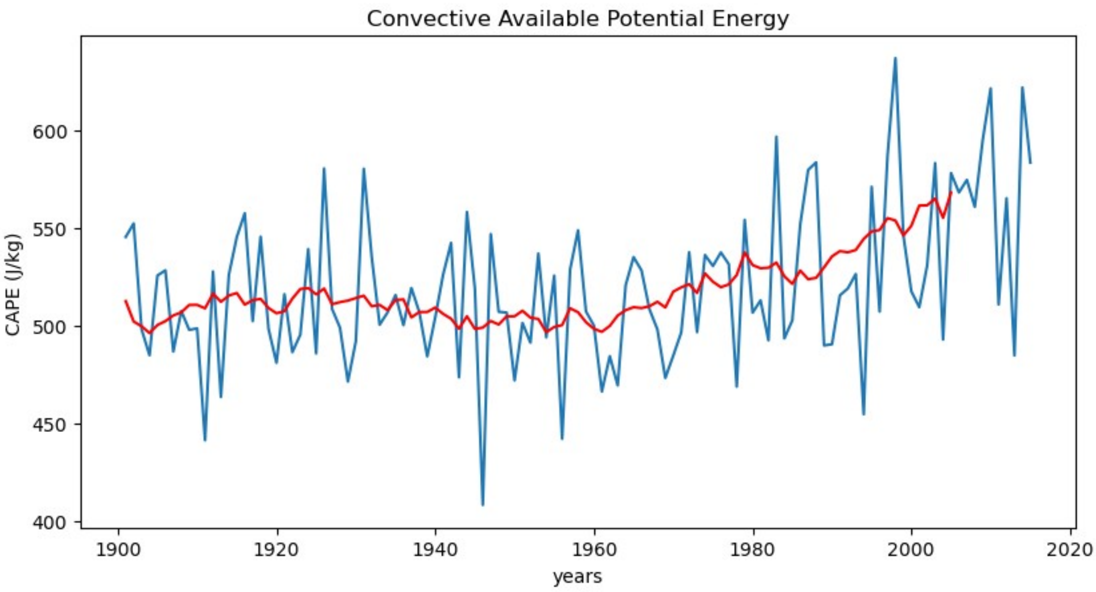
Convective Available Potential Energy
CAPE (J/kg)
600
550
500
450
400
1900
1920
1940
1960
1980
2000
2020
years

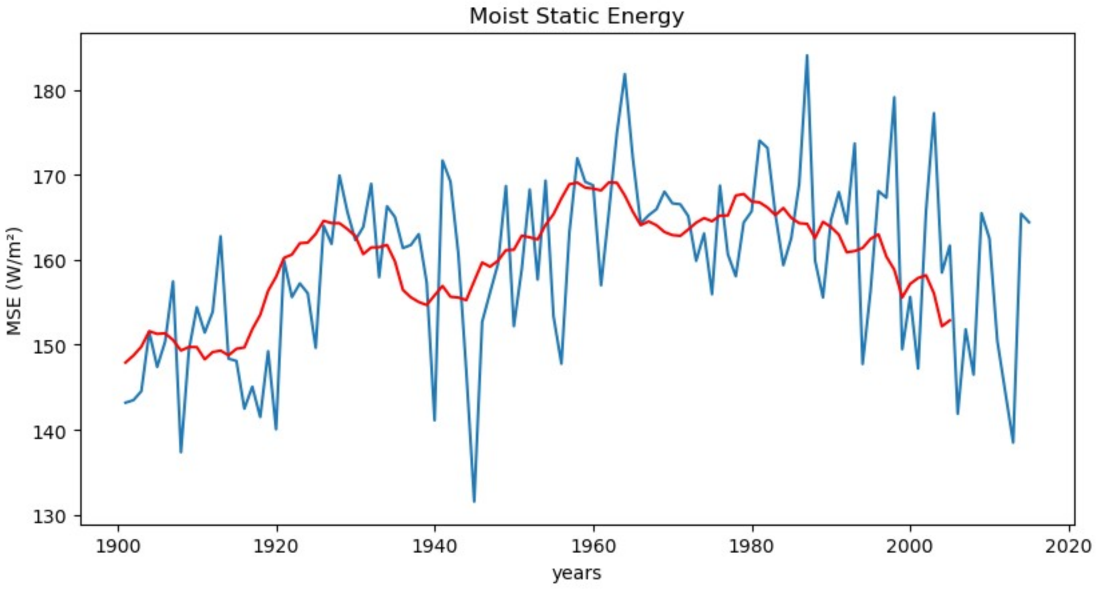
Moist Static Energy
MSE (W/m²)
180
170
160
150
140
130
1900
1920
1940
1960
1980
2000
2020
years

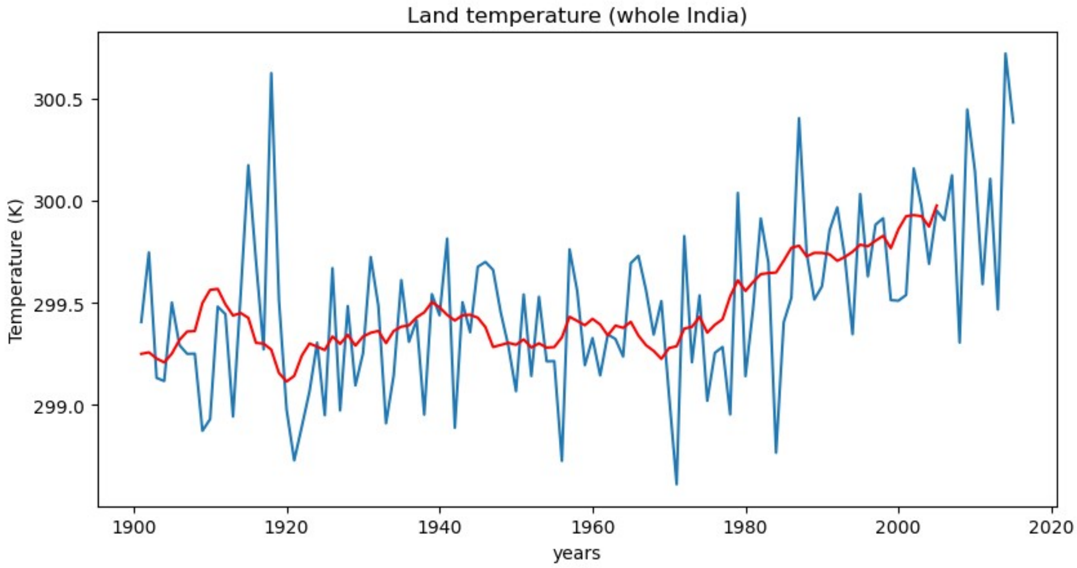
Land temperature (whole India)
Temperature (K)
300.5
300.0
299.5
299.0
1900
1920
1940
1960
1980
2000
2020
years

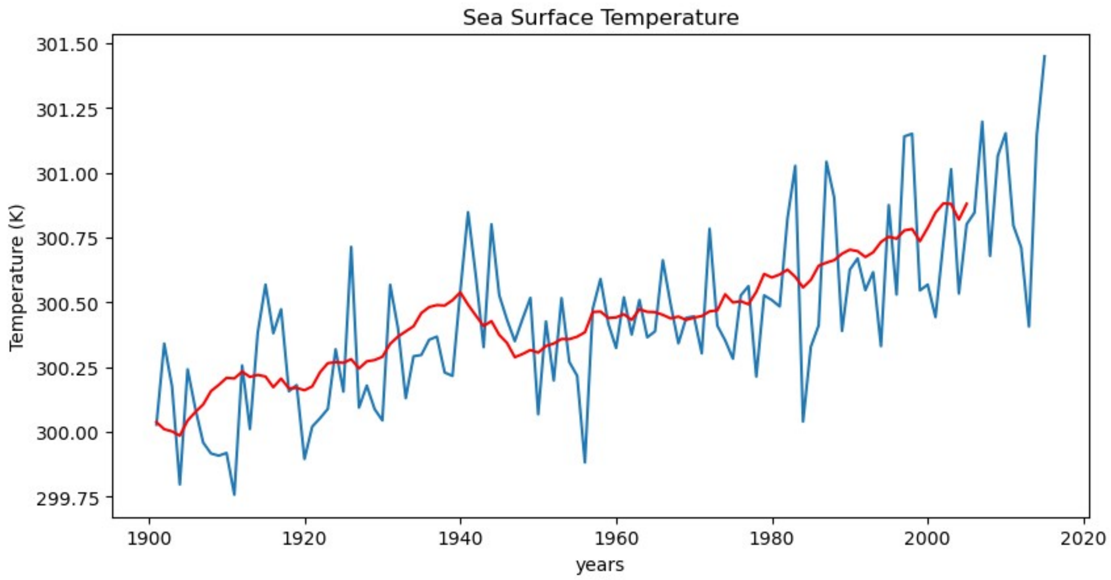
Sea Surface Temperature
Temperature (K)
years
301.50
301.25
301.00
300.75
300.50
300.25
300.00
299.75
1900
1920
1940
1960
1980
2000
2020

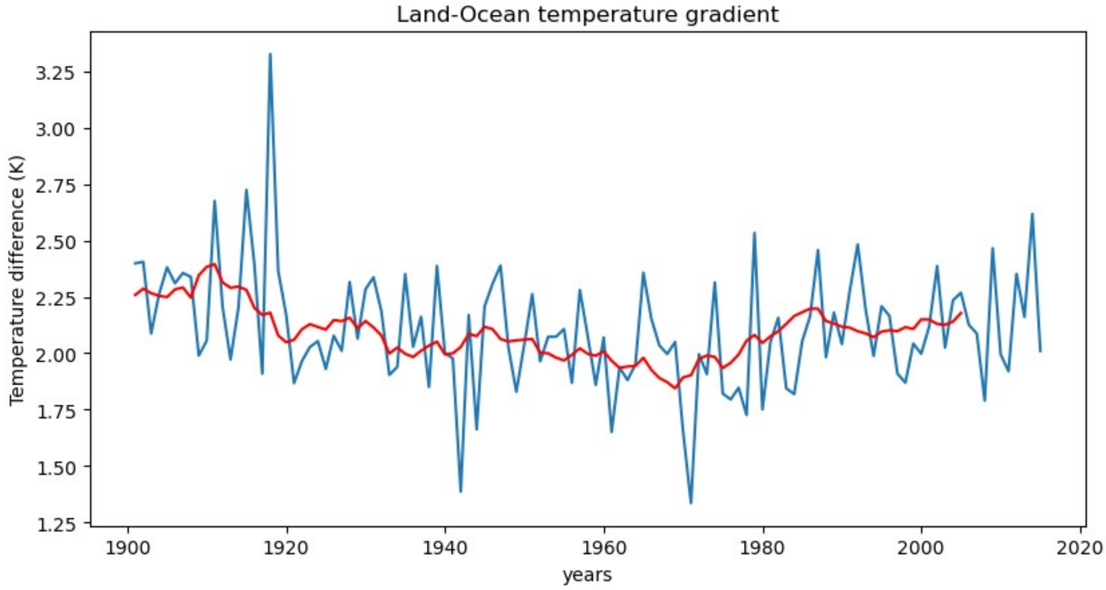
Land-Ocean temperature gradient
Temperature difference (K)
years
3.25
3.00
2.75
2.50
2.25
2.00
1.75
1.50
1.25
1900
1920
1940
1960
1980
2000
2020

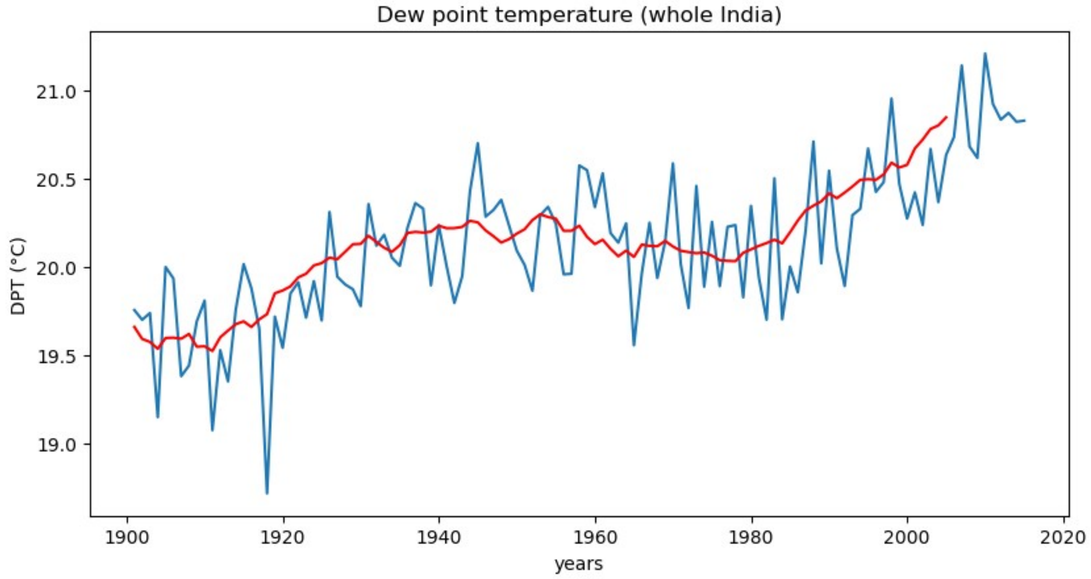
Dew point temperature (whole India)
DPT (°C)
years
21.0
20.5
20.0
19.5
19.0
1900
1920
1940
1960
1980
2000
2020

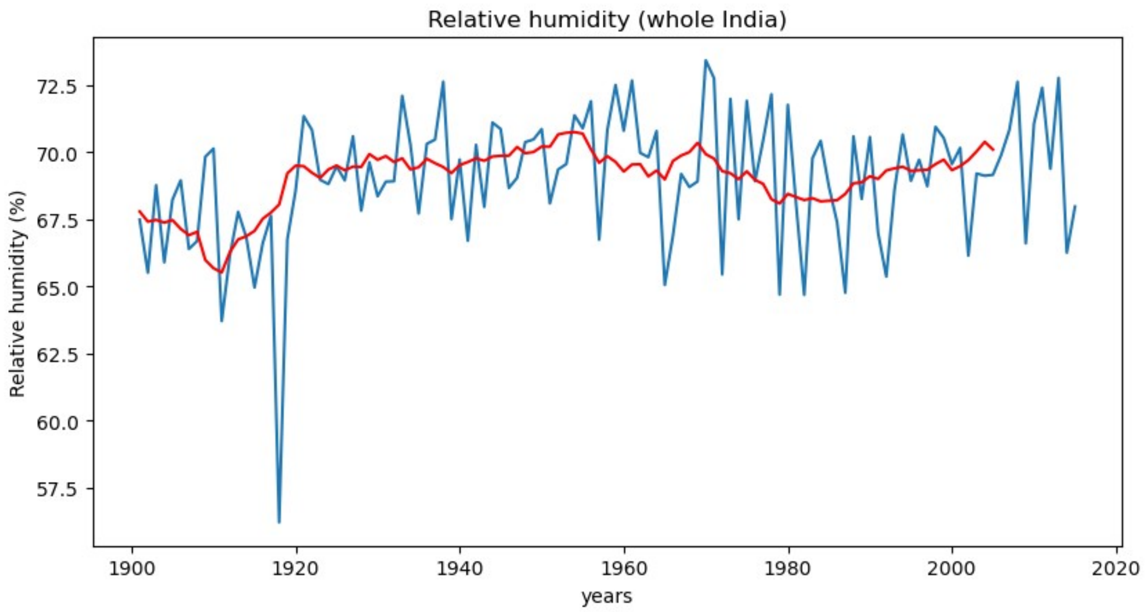

Fig. S4 | Input features for the Random forest regression. The time series of the 17 input features used in the random forest regression model are presented. The yearly values are plotted in blue and the 10-year moving averages used in the regression are in red. For the LULC data, we used simple linear interpolation between the different observation data points. For the temperature, dew point temperature, relative humidity and specific humidity, the values averaged over the whole India are shown here, whereas we used regional average in the random forest regression.
